# Supplementary material for: A map of directional genetic interactions in a metazoan cell
Source: eLife. 2015 Mar 6;4:e05464. doi: 10.7554/eLife.05464 (PMC4384530; doi:10.7554/eLife.05464)
Supplement: Source code 1. — Documented source code for reproducing all figures and tables. DOI: http://dx.doi.org/10.7554/eLife.05464.025 [file elife05464s007.pdf]

Experimental data and documented source code for the paper

A Map of Directional Genetic Interactions in a Metazoan Cell

Bernd Fischer, Thomas Sandmann, Thomas Horn, Maximilian Billman, Varun Chaudhary, Wolfgang Huber, and Michael Boutros

eLife, in press, http://www.elifesciences.org.

Bernd Fischer

German Cancer Research Center (DKFZ),
Heidelberg, Germany
b.fischer@dkfz.de

March 24, 2015

Contents

1 Introduction 2

2 Data access 2

3 Re-analysis of a genetic interaction screen and experimental design 3

4 Selection of target and query genes 5
- 4.1 Target gene selection . . . 5
- 4.2 Query gene selection . . . 5

5 Image processing 6
- 5.1 Image segmentation and feature extraction . . . 6
- 5.2 Transformation and scaling . . . 8
- 5.3 Single knockdown examples . . . 8

6 Quality control 10
- 6.1 Quality control of features . . . 10
- 6.2 Quality control of dsRNA . . . 11
- 6.3 Discussion of feature and target gene reagent quality control . . . 13
- 6.4 Comparison to Rohn et al. . . . 13

7 Feature selection 15

8 Pairwise interaction scores 17
- 8.1 Double knockdown examples . . . 17
- 8.2 Main result table . . . 19
- 8.3 Comparison of main effects across different phenotypes . . . 20
- 8.4 Comparison of main effects across batches . . . 22
- 8.5 Comparison of pairwise interaction scores across different phenotypes . . . 24
- 8.6 Numbers of genetic interactions . . . 26

9 Multi-phenotype genetic interaction cube **27**
9.1 Heatmap of genetic interaction cube 28
9.2 Comparison to DPiM 29

10 Genetic interaction landscape **30**
10.1 Correlation graph 30

11 Directional epistatic interactions **35**
11.1 Inference of a directional, epistatic interactions 35
11.2 A table of all directional interactions 39
11.3 An directional epistatic network for mitosis. 39
11.4 Subgraphs of the directional network 41
11.5 Directional interactions of recurrently mutated genes 42

12 Session info **43**

1 Introduction

This document is associated with the *Bioconductor* package *DmelSGI*, which contains the data and the *R* code for the statistical analysis presented in the paper

> *A Map of Directional Genetic Interactions in a Metazoan Cell*
> Bernd Fischer, Thomas Sandmann, Thomas Horn, Maximilian Billman, Varun Chaudhary, Wolfgang Huber, and Michael Boutros
> eLife, in press, http://www.elifesciences.org.

In Section 2, the access to the data is described. The *R* code within each section can be executed independently of the other sections. Intermediate results from each section are available in the form of *R* data objects.

To install the *DmelSGI*, please start a current version of *R* and type

```
source("http://bioconductor.org/biocLite.R")
biocLite("DmelSGI")
```

Some convenience functions for calling sub-vignettes.

```
fpath <- function(d) { file.path(opts_knit$get("output.dir"), "result", d,"") }
```

2 Data access

The matrix of statistical genetic interactions can be loaded by

```
library("DmelSGI")
##
## Attaching package:  'DmelSGI'
##
## The following object is masked _by_ '.GlobalEnv':
##
##    fpath
data("Interactions", package="DmelSGI")
```

The element `Interactions$piscore` of this data object is a 3-dimensional array of π-scores, `Interactions$padj` is a 3-dimensional array of BH-adjusted p-values, and `Interactions$Anno` contains annotation for target genes, query genes, and phenotypes. You can type

```
? Interactions
```

to see the documentation of this data object.

3 Re-analysis of a genetic interaction screen and experimental design

The choice of the target–query experimental design was based on our re-analysis of a symmetric genetic interaction matrix of 93 genes described by [1].

```
library("DmelSGI")
library("RNAinteractMAPK")

basedir = getBaseDir()
resultdir = file.path( basedir, "result", "ReanalysisOfHornEtAl")
dir.create(resultdir, recursive = TRUE,showWarnings=FALSE)
```

Load the matrix with pairwise interaction scores of the Ras-signaling genetic interaction screen from the *Bioconductor* package *RNAinteractMAPK* [1].

```
data("Dmel2PPMAPK", package="RNAinteractMAPK")
print(Dmel2PPMAPK)

## RNA interaction screen
## Nr of template reagents:    384
## Nr of query reagents:        192
## Nr of experiments in screen:  36864
## Nr of channels:               3
## Nr of screens:               3

PI <- getData(Dmel2PPMAPK, type="pi", format="targetMatrix", screen="mean",
              withoutgroups = c("pos", "neg"))[,,1,]
```

Divide by the standard deviation to normalize each genetic interaction profile per phenotype.

```
for (j in 1:dim(PI)[2]) {
  for (k in 1:dim(PI)[3]) {
    PI[,j,k] = PI[,j,k] / (sqrt(sum(PI[,j,k] * PI[,j,k]) / (dim(PI)[2]-1)))
  }
}
```

The query genes are ordered in a greedy manner, such that the genetic interaction profiles of the first genes describe most of the total variance in the whole genetic interaction screen. *warning: The next code-chunk requires a long run-time. Do not run the code, the result is hard coded afterwards.*

```
Selected = c()
Selected = c(1,2,3)
R = 1:dim(PI)[1]
Res = PI
openVar = rep(-1,dim(PI)[1]+1)
openVar[1] = sum(Res * Res) / (dim(PI)[1]*(dim(PI)[2]-1)*dim(PI)[3])
for (i in 1:dim(PI)[1]) {
  H = rep(100000000.0,length(R))
  for (j in 1:length(R)) {
    cat("i=",i," j=",j,"\n")
    k=1:3
    A = PI[,c(Selected[seq_len(i-1)],R[j]),k,drop=FALSE]
    dim(A) = c(dim(A)[1],prod(dim(A)[2:3]))
    B = PI[,-c(Selected[seq_len(i-1)],R[j]),k,drop=FALSE]
    dim(B) = c(dim(B)[1],prod(dim(B)[2:3]))
```

```
    Res = matrix(0.0, nr=dim(PI)[1],nc=ncol(B))
    for (z in 1:ncol(B)) {
      model = lm(B[,z]~A+0)
      Res[,z] = model$residuals
    }
    H[j] = sum(Res * Res) / (dim(PI)[1]*(dim(PI)[2]-1)*dim(PI)[3])
  }
  M = which.min(H)
  cat("selected: ",R[M],"\n")
  Selected = c(Selected, R[M])
  openVar[i+1] = H[M]
  R = R[-M]
}
```

To avoid long runtimes, the result from the previous code chunk is hard coded in the following code chunk.

```
openVar = c(1, 0.584295886914632, 0.49354448724904, 0.440095163032832,
  0.37969110256306, 0.330693818887106, 0.28896777328302, 0.26144276377077,
  0.24550380797587, 0.212282252772014, 0.19041097617251, 0.16974901306481,
  0.15642204582756, 0.141467140253324, 0.12781027389229, 0.11609596000734,
  0.10374891651534, 0.093268306952119, 0.08446425055463, 0.07404659630757,
  0.06599890651265, 0.057244319680828, 0.04944008500553, 0.04161924747819,
  0.03515950952616, 0.028667487889006, 0.02313772533424, 0.01727915218118,
  0.01282727545013, 0.007910401967279, 0.00357968641756,
  0, 0, 0, 0, 0, 0, 0, 0, 0, 0, 0, 0, 0, 0, 0, 0, 0, 0, 0, 0, 0,
  0, 0, 0, 0, 0, 0, 0, 0, 0, 0, 0, 0, 0, 0, 0, 0, 0, 0, 0, 0, 0,
  0, 0, 0, 0, 0, 0, 0, 0, 0, 0, 0, 0, 0, 0, 0, 0, 0, 0, 0, 0, 0
)

Selected = c(1, 2, 3, 16, 47, 9, 48, 63, 22, 74, 77, 53, 31, 27, 60, 6,
15, 93, 5, 82, 67, 45, 91, 7, 30, 25, 59, 13, 55, 61, 54, 35,
84, 4, 1, 2, 3, 8, 10, 11, 12, 14, 17, 18, 19, 20, 21, 23, 24,
26, 28, 29, 32, 33, 34, 36, 37, 38, 39, 40, 41, 42, 43, 44, 46,
49, 50, 51, 52, 56, 57, 58, 62, 64, 65, 66, 68, 69, 70, 71, 72,
73, 75, 76, 78, 79, 80, 81, 83, 85, 86, 87, 88, 89, 90, 92)
```

Barplot showing the fraction of explained variance by the first n query genes.

```
N = 1:dim(PI)[1]
bp = barplot(100.0*(1-openVar[N+1]),ylim=c(0,100),
             ylab="explained variance [in %]",xlab="number query genes",
             cex.axis=1.5,cex.lab=1.5)
axis(side=1,at=bp[N %% 10 == 0],labels=(N)[N %% 10 == 0],cex.axis=1.5)
```

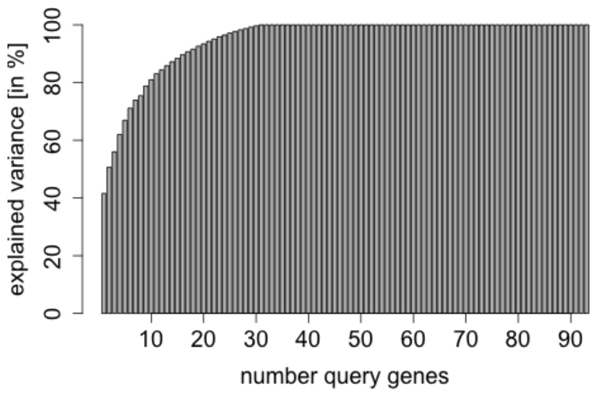

This analysis revealed that the full information content could be approximated by restricting one dimension of the

matrix to a subset of genes. 17 query genes explain 90% variance. 22 query genes explain 95% variance. Thus, 10 to 20 *suitably selected* query genes are sufficient to explain almost all of the variance in this data. A similar result was reported by [2] for yeast interaction matrices.

4 Selection of target and query genes

4.1 Target gene selection

```
library(DmelSGI)

basedir = getBaseDir()
resultdir = file.path( basedir, "result", "QueryGeneSelection")
dir.create(resultdir, recursive = TRUE,showWarnings=FALSE)

data("datamatrix", package="DmelSGI")
```

The experiment assayed all pairwise combinations of 1367 target genes times 72 query genes. The target genes were chosen according to the following criteria.

- Coverage by ≥ 5 reads in an RNA-seq data set [3] of the cell line's transcriptome.
- Existence of an orthologous gene in H. sapiens, M. musculus, C. elegans or S. cerevisiae.
- Annotation with one or more of the following Gene Ontology terms or Panther pathway names:
  - DNA metabolism (BP00034, GO:0006259)
  - mRNA transcription (BP00040, GO:0006366)
  - nucleoside, nucleotide and nucleic acid transporter (BP00058, GO:0015931)
  - chromatin packaging and remodeling (BP00273, GO:0006333)
  - oncogenesis (BP00281)
  - cell cycle (BP00203, GO:0007049)
  - nucleic acid binding (MF00042, GO:0003676)
  - transcription factor (MF00036, GO:0030528)
  - cell cycle (P00013)
  - DNA replication (P00017)
  - general transcription regulation (P00023)

  Genes annotated with the following categories were excluded:
  - tRNA metabolism (BP00054 , GO:0006399)
  - histones (MF00063)
  - mRNA processing factors (MF00065, GO:0003729)
  - translation factors (MF00071, GO:0008135)
  - ribosomal proteins (MF00075,GO:0003735)
  - RNA-methyltransferase (MF00054, GO:0008173)

4.2 Query gene selection

To choose the query genes, we performed an initial screen, using the same setup that was going to be used for the main experiment, on single-dsRNA perturbations of all target genes.

```
library("DmelSGI")

data("SKDdata",package="DmelSGI")
data("datamatrix",package="DmelSGI")
```

A principal component analysis of a single knock-down experiment.

```
D = apply(SKDdata$D[ ,,1,],
          c(1,3), mean, na.rm=TRUE)
PCA = princomp(D)
```

The selected query genes are colored red, the other genes gray. Genes are ordered, such that the red points are plotted last.

```
col = ifelse(datamatrix$Anno$target$TID %in% datamatrix$Anno$query$TID,
             "red","gray80")
I = order(datamatrix$Anno$target$TID %in% datamatrix$Anno$query$TID)
S = PCA$scores
S = S[I,]
col = col[I]
```

Resulting multivariate phenotypes were visualised through scatter plots of the data projected on the first five principal components.

```
par(mar=c(0.2,0.2,0.2,0.2))
pairs(S[,1:5],pch=20,cex=0.7,col=col)
```

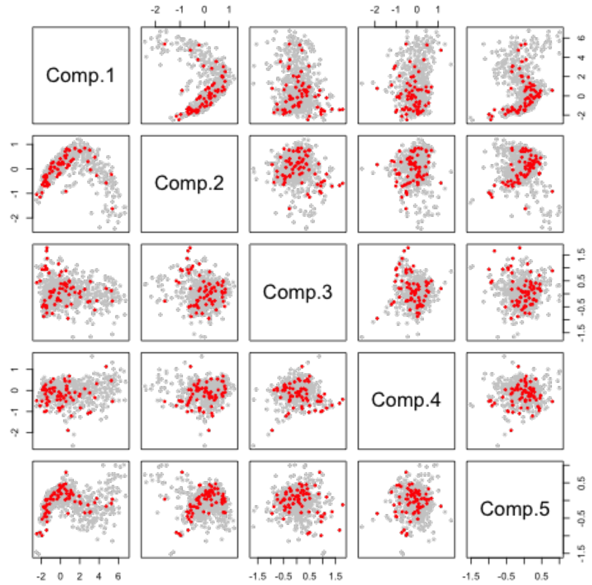

We selected 72 genes that had a moderately strong effect as assessed by visual analysis of the images, and in addition we required that they were approximately uniformly dispersed in the space of phenotypes as assessed in the principal component projection. The selected query genes are colored red.

5 Image processing

5.1 Image segmentation and feature extraction

```
library("DmelSGI")
library("RColorBrewer")

basedir = getBaseDir()
resultdir = file.path( basedir, "result", "ImageProcessing")
dir.create(resultdir, recursive = TRUE,showWarnings=FALSE)

data("datamatrix",package="DmelSGI")
data("Features",package="DmelSGI")
```

Image analysis and features extraction was adapted from previously described methods [4, 5, 6, 7], using the R package *EBImage* [4]. In the following, we use the term *experiment* to denote a single well in a 384-well plate, corresponding to a cell population subjected to either a pair of dsRNAs, or to control reagents. Of each experiment,

two images were analysed, one at 4x magnification and one at 10x magnification.

4x-images Nuclei were segmented and identified separately in the DAPI and pH3 channels. Regions containing nuclei were separated from the image background by adaptive thresholding (window width 4 pixels). Nuclei were identified by local maximum search on the intensities within nuclei-containing regions, resulting in seeds. Seeds were extended to estimates of the whole nucleus area by the propagation algorithm of [8].

The set of nuclei detected in the pH3 channel was matched to the set of nuclei detected in the DAPI channel by searching the nearest neighbor. The algorithm employed the fact that the mitotic nuclei visible in the pH3 channel were a sparse subset of all nuclei visible in the DAPI channel. Matches were accepted if the distance between the centers of the nuclei in the two channels was ≤ 5 pixels.

84 features were extracted. These included:

1. Number of nuclei in the DAPI channel, number of nuclei in the pH3 channel and absolute number and fraction of nuclei in the DAPI channel with a matching nucleus in the pH3 channel.
2. Mean and standard deviation of area and fluorescence intensity in both channels.
3. Local cell density was estimated with a kernel density estimator using a Gaussian kernel on different scales ($\sigma \in \{1, 2, 4, 8, 16, 32, 64, 128$ pixels$\}$). The mean of this density estimate evaluated on the center of cells was used as a feature.
4. The ratio of the local cell density features for $\sigma = 8$ pixels and $\sigma = 128$ pixels.
5. 3%, 10%, 25%, 50%, 75%, 90%, and 97% quantiles of area and fluorescence intensity.
6. Histograms of fluorescence intensity and area. The histogram bin sizes were preset based on visual inspection of data from prior control experiments.

Features under items 1 and 2 were extracted once for all cells in the experiment, and once for cells within a radius of 900 pixels around the center of the image, to avoid shading effects. The features under item 3 tended to correlate with the total number of cells, while the feature of item 4 was chosen to be approximately independent of the total number of cells and to reflect potential higher-order clustering effects. The full list of features is generated in Section 6.1.

10x-images Nuclei were segmented and identified separately in the DAPI and pH3 channels. Regions containing nuclei were separated from the image background by adaptive thresholding (window width 10 pixels) and subsequent morphological opening. Nuclei identified in the pH3 channel were mapped to the nuclei extracted from the DAPI channel by maximum overlap. The nucleus regions extracted from the DAPI channel were extended to the cell body by the propagation algorithm of [8] using the fluorescence levels in the α-tubulin channel.

244 features were extracted. These included:

1. Moments (length of the major axis and eccentricity).
2. Shape features (area, perimeter, minimum radius, mean radius and maximum radius).
3. Basic features (mean, standard deviation, median deviation, 1%, 5%, 50%, 95%, 99% quantiles of intensity)
4. Haralick features [9] on two scales to quantify texture.

These features were computed for the nuclei segmentation from the DAPI channel and for cell segmentation from the α-tubulin channel. The intensity values considered were taken from the DAPI channel, the α-tubulin channel, and from a synthetic third channel which was computed by the cross-correlation of the DAPI and α-tubulin intensities. The pH3 intensities were used to classify the cells into two classes, mitotic and non-mitotic. To obtain features per experiment, the mean of the single cells features was computed separately for the mitotic and non-mitotic cells. The full list of features is generated in Section 6.1.

A 5D data cube After feature extraction, the data were represented in a 5-dimensional array with dimensions

- 1367 target genes,
- 2 dsRNA designs per target gene,
- 72 query genes,
- 2 dsRNA designs per query gene,
- 328 features.

5.2 Transformation and scaling

Previous genetic interaction screens that were based on a quantitative cell viability phenotype were analysed using a multiplicative interaction model [10, 11, 12, 13, 14]. For model fitting, it is then convenient to transform the data to a logarithmic scale. Motivated by [1, 15], we adapted this approach to all features considered here. Since some features had a non-positive range of values, instead of the logarithm we applied a generalized logarithm transform [16]

$$f(x;c) = \log\left(\frac{x + \sqrt{x^2 + c^2}}{2}\right). \tag{1}$$

This family of functions has one parameter c. For $c = 0$, the function is equivalent to an ordinary logarithm transformation. For $c > 0$, the function is smooth for all values of x (including 0 and negative values), avoiding the singularity of the ordinary logarithm at $x = 0$, but still approximately equivalent to the ordinary logarithm for $x \gg c$. See the following Figure. For each feature, we chose c to be the 3%-quantile of the feature's empirical distribution.

```
px = seq(-1.5, 9, length.out=200)
trsf = list(
  log = function(x) log(ifelse(x>0, x, NA_real_)),
 glog = function(x, c=1) log( (x+sqrt(x^2+c^2))/2 ))
colores = c("#202020", "RoyalBlue")

matplot(px, sapply(trsf, do.call, list(px)), type="l", lty=c(2,1), col=colores, lwd=2.5,
        ylab="f(x)", xlab="x")
legend("bottomright", fill=colores, legend=names(trsf))
```

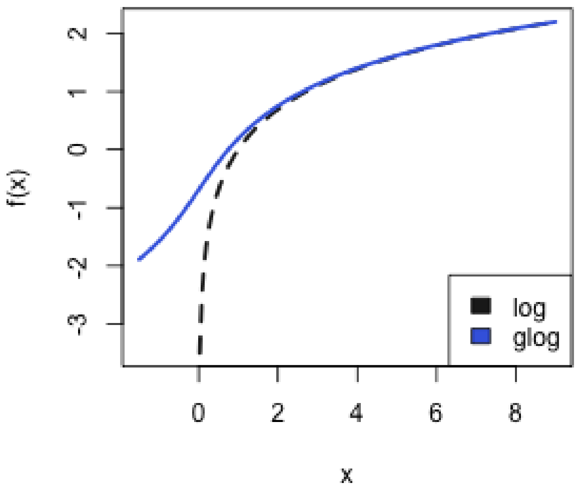

After transformation, to take account of the fact that the data range of the different features was different, data were centered and scaled separately for each feature. Center and scale were computed as the median and median absolute deviation, respectively.

In the following, we will refer to the array of transformed, centered and scaled values as D_{ijklm}, with the indices i, j, k, l, m counting over the 5 dimensions listed above.

5.3 Single knockdown examples

```
data("mainEffects",package="DmelSGI")
```

Average main effects (single knockdown effects) over dsRNA designs and batches and add a column for control (Fluc).

```
Main = apply(mainEffects$target,c(1,4),mean,na.rm=TRUE)
m = apply(Main,2,mad,center=0.0)
for (i in 1:dim(Main)[2]) {
  Main[,i] = Main[,i] / m[i]
}
Main = rbind(Main,Fluc=c(0.0,0.0,0.0))
```

```
ylim = range(Main[c("Fluc","ida","stg","Arpc1"),1:3])
col = brewer.pal(4,"Pastel1")
```

```
par(mar=c(0.2,2,2,0.2))
barplot(Main["Fluc",c(1:3,13)],main="Fluc", col=col,ylim=ylim)
abline(h=0.0)

barplot(Main["ida",c(1:3,13)],main="ida", col=col,ylim=ylim)
abline(h=0.0)

barplot(Main["stg",c(1:3,13)],main="stg", col=col,ylim=ylim)
abline(h=0.0)

barplot(Main["Arpc1",c(1:3,13)],main="Arpc1", col=col,ylim=ylim)
abline(h=0.0)

plot(-10000,xaxt="n",yaxt="n",bty="n",xlim=c(0,1),ylim=c(0,1),xlab="",ylab="")
legend("topleft",c("nr cells","MI","area","eccent."),fill=brewer.pal(4,"Pastel1"))
```

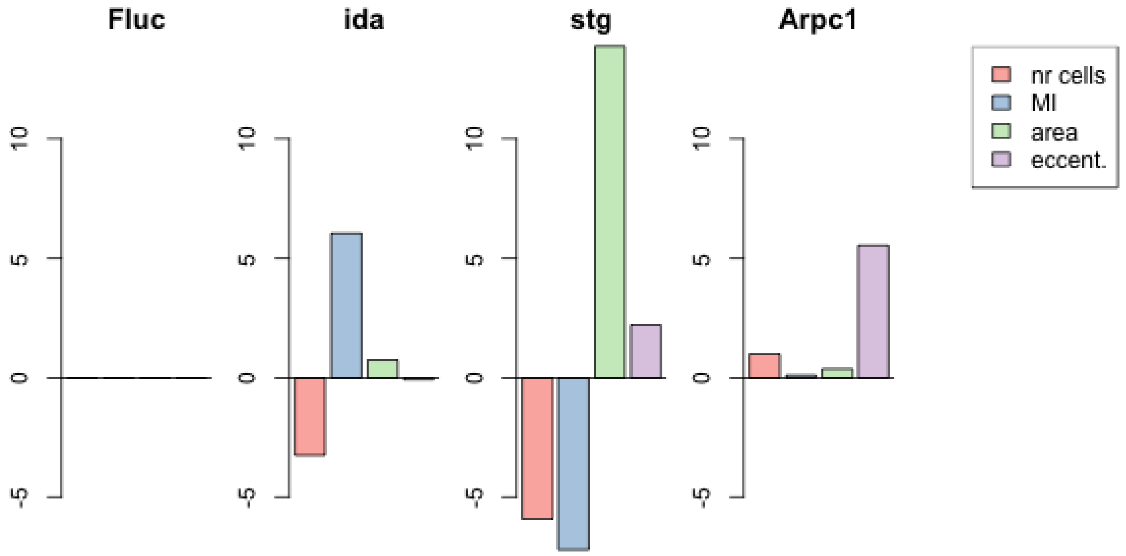


```
barplot(Main[c("Fluc","RasGAP1"),1],col=brewer.pal(3,"Pastel1")[1],
        ylab=c("cell number","z-score"),yaxp=c(0,1,2),las=2)
```

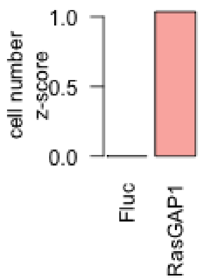

6 Quality control

```
library("beeswarm")
library("RColorBrewer")
library("DmelSGI")
library("hwriter")
library("xtable")

basedir = getBaseDir()
resultdir = file.path( basedir, "result", "QualityControl")
dir.create(resultdir, recursive = TRUE,showWarnings=FALSE)

data("datamatrix", package="DmelSGI")
```

6.1 Quality control of features

To control for the quality of each feature, its reproducibility over replicate measurements was assessed. To this end, we employed the two independent dsRNA designs in the role of replicates (note that this make our quality control more stringent than if we were using regular replicates, since the differences in the dsRNA reagents can introduce additional variation). For each feature m, we computed the two vectors $\mathbf{v}_m^1$ and $\mathbf{v}_m^2$,

$$\mathbf{v}_m^l = D_{\cdot\,-\,\cdot\,lm} \qquad \text{for } l = 1, 2. \tag{2}$$

Here, the notation $-$ indicates averaging over an index, and the notation $\cdot$ indicates extraction of the whole subspace spanned by this index; thus, $\mathbf{v}_m^l$ is a vector with $1367 \times 72 = 9.8424 \times 10^4$ elements. We then computed the correlation coefficient ρ_m between $\mathbf{v}_m^1$ and $\mathbf{v}_m^2$.

```
data("qualityControlFeature", package="DmelSGI")
data("Features", package="DmelSGI")
```

```
Fcor = qualityControlFeature$correlation
Fcor = Fcor[order(-Fcor)]
```

```
par(mar=c(4.1,4.1,1,1))
plot(Fcor,pch=19,xlab="features",
     ylab=c("correlation of phenotype","between replicates"),
     ylim = range(qualityControlFeature$correlation,finite=TRUE),
     xlim=c(0,sum(is.finite(Fcor))),cex.lab=1.3, cex.axis=1.3)
abline(h=0.6)
```

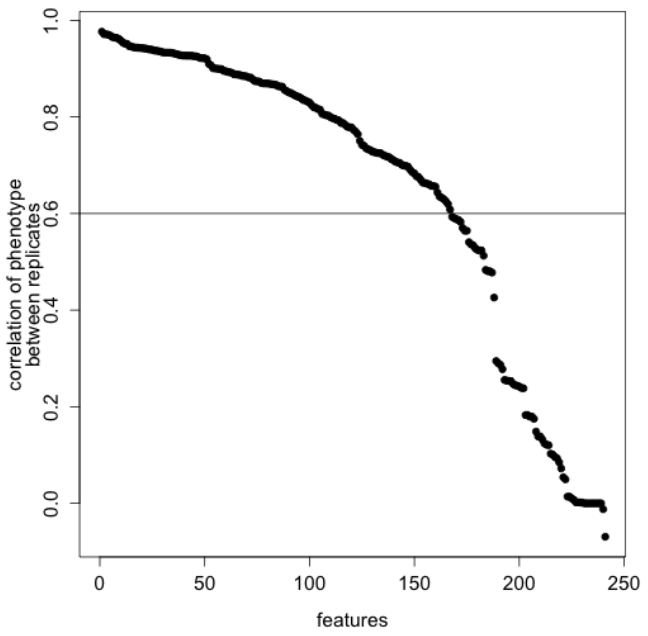

Some correlation coefficients could not be computed because the vector elements did not show any variation. We selected features for subsequent analysis that fulfilled the following two criteria:

- $\rho_m \geq 0.6$,
- Less than 1% of values in $D_{\cdot\cdot\cdot\cdot m}$ undefined.

162 features fulfilled this criterion.

```
data("Features", package="DmelSGI")
data("qualityControlFeature", package="DmelSGI")
```

A table of all features is written to a text file.

```
Features = cbind(Features, QC=ifelse(qualityControlFeature$passed, "passed", "failed"),
                 name = hrNames(row.names(Features)))
write.table(Features, file=file.path(resultdir,"FeatureTable.txt"), sep="\t", quote=FALSE)
```

The head of the table of all features looks like this:

Table 1: Features extracted from images

	mag	summary	mask	channel	set	type	param	QC	name
4x.count	4x	nrNuclei	nucleus	DAPI	M			passed	cell number
4x.countpH3	4x	nrNuclei	mitoticNuclei	pH3	M			passed	number of mitotic objects
4x.isMitotic	4x	nrNuclei	nucleus	DAPI	M			passed	number of mitotic nuclei
4x.ratioMitotic	4x	mitoticRatio	nucleus	DAPI	M			passed	mitotic index
4x.areaNuc	4x	mean	nucleus	DAPI	M	area		passed	4x.areaNuc
4x.areaNucSD	4x	stddev	nucleus	DAPI	M	area		passed	4x.areaNucSD
4x.areapH3	4x	mean	mitoticNuclei	pH3	M	area		passed	4x.areapH3

6.2 Quality control of dsRNA

```
data("qualityControlGene", package="DmelSGI")
```

To detect cases where the dsRNA reagents for our target genes had off-target effects, we compared the phenotypic profiles of the two dsRNA designs for each target gene. To this end, we computed the vectors

$$\mathbf{w}_i^j = D_{ij\,\cdot\,-\,\cdot} \quad (3)$$

using only those features that passed the quality filter described in Section 6.1. We then computed the correlation $\tilde{\rho}_i$ between $\mathbf{w}_i^1$ and $\mathbf{w}_i^2$. Target gene i was selected for subsequent analysis if $\tilde{\rho}_i \geq 0.7$. 1389 genes passed this quality control criterion.

```
par(mar=c(4.1,4.5,1,1))
Sample = which(qualityControlGene$Annotation$group == "sample")
corGene = qualityControlGene$correlation[Sample]
corGene = corGene[order(-corGene)]
plot(corGene,
     pch=19,
     xlab="targeted genes",
     ylab=c("cor of phenotypic profile","between dsRNA designs"),
     cex.lab=1.3, cex.axis=1.3)
abline(h=0.7)
```

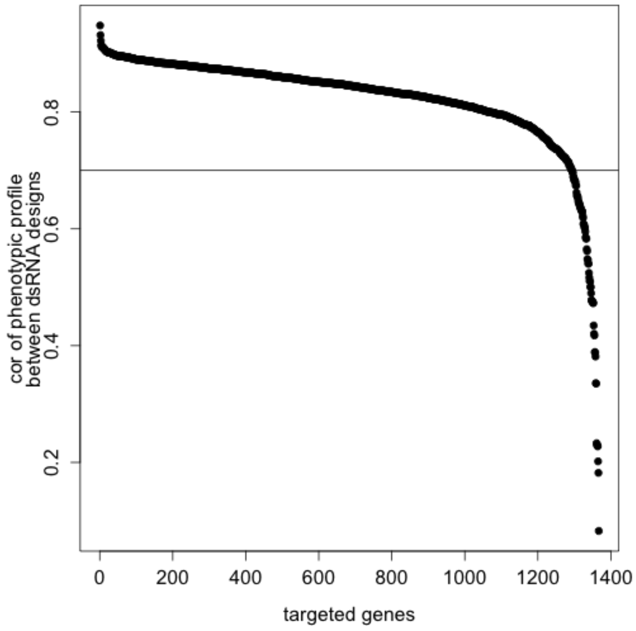

A table of all genes that passed quality control is written to a text file.

```
data("qualityControlGene", package="DmelSGI")
PassedSamples = which((qualityControlGene$Annotation$group == "sample")
                      & (qualityControlGene$passed))
A = qualityControlGene$Annotation
A$cor = qualityControlGene$correlation
A = A[,c("TID", "Symbol", "cor", "Name")]
A = A[PassedSamples,]
A = A[order(A$cor),]
A$cor = sprintf("%0.2f", A$cor)
write.table(A, file=file.path(resultdir,"PassedGenes.txt"), sep="\t",
            quote=FALSE,row.names=FALSE)
```

The head of the table of genes that passed quality control looks like this:

The data remaining after these quality control steps were represented in a 5-dimensional array Y_{ijklm} with dimensions

- 1293 target genes,
- 2 dsRNA designs per target gene,
- 72 query genes,
- 2 dsRNA designs per query gene,
- 162 features.

Table 2: List of genes that passed the quality control

	TID	Symbol	cor	Name
99	FBgn0263968	nonC	0.70	no-on-and-no-off transient C
248	FBgn0030990	CG7556	0.70	
528	FBgn0011725	twin	0.70	twin
726	FBgn0010762	simj	0.71	simjang
584	FBgn0053182	Kdm4B	0.71	Histone demethylase 4B
1519	FBgn0010602	lwr	0.71	lesswright
546	FBgn0036534	Dcp2	0.71	Decapping protein 2

6.3 Discussion of feature and target gene reagent quality control

The correlation-based criteria used in Sections 6.1 and 6.2 are biased towards features and reagents that report biological signal in a large fraction of measurements. In principle it is possible that features or reagents that did not pass these criteria could still contain important information, e. g. if their data is only reporting signal for a few rare interactions and otherwise simply fluctuates around a baseline value. In this paper, such features and reagents were excluded, however, their data might still be useful for additional analyses.

6.4 Comparison to Rohn et al.

We compared the extracted features to phenotypes reported by [17].

```
data("SKDdata", package="DmelSGI")
data("mainEffects", package="DmelSGI")
data("RohnEtAl", package="DmelSGI")

RohnEtAl = RohnEtAl[which(RohnEtAl$Computed.Target %in% SKDdata$Anno$target$TID),]
I = match(RohnEtAl$Computed.Target, SKDdata$Anno$target$TID)

RohnEtAlanno = RohnEtAl[,1:3]
RohnEtAl = as.matrix(RohnEtAl[4:29])
D = apply(SKDdata$D[I,,,],c(1,4), mean, na.rm=TRUE)

i=3; j=2
anova(lm(D[,i] ~ RohnEtAl[,j]))

## Analysis of Variance Table
##
## Response: D[, i]
##                  Df Sum Sq Mean Sq F value    Pr(>F)
## RohnEtAl[, j]   1 1.8156 1.81564  38.165 5.828e-09 ***
## Residuals     150 7.1361 0.04757
## ---
## Signif. codes:  0 '***' 0.001 '**' 0.01 '*' 0.05 '.' 0.1 ' ' 1

beeswarm(D[,i] ~ RohnEtAl[,j],pch=20, xlab=c(colnames(RohnEtAl)[j],"Rohn et al."),
         ylab=dimnames(D)[[2]][i])
```

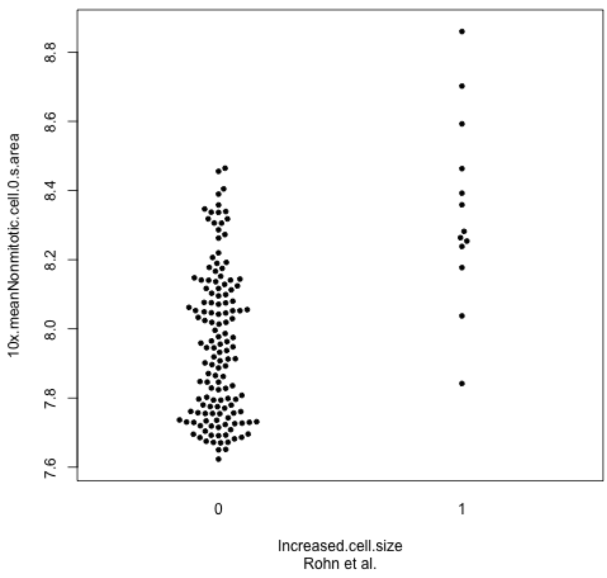


```
i=13; j=5
anova(lm(D[,i] ~ RohnEtAl[,j]))
```

```
## Analysis of Variance Table
##
## Response: D[, i]
##                  Df   Sum Sq   Mean Sq F value    Pr(>F)
## RohnEtAl[, j]    1 0.020031 0.0200306  13.577 0.0003192 ***
## Residuals      150 0.221300 0.0014753
## ---
## Signif. codes:  0 '***' 0.001 '**' 0.01 '*' 0.05 '.' 0.1 ' ' 1
```

```
beeswarm(D[,i] ~ RohnEtAl[,j],pch=20, xlab=c(colnames(RohnEtAl)[j],"Rohn et al."),
         ylab=dimnames(D)[[2]][i])
```

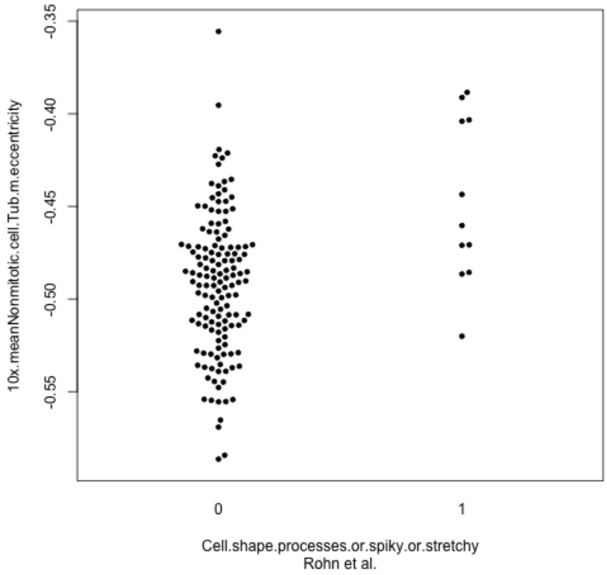

7 Feature selection

To select a subset of non-redundant features, we performed dimension reduction by stepwise feature selection. Features were selected according to the stability of their non-redundant contribution to the overall data set, as described in this section.

We started by selecting three features, number of cells, fraction of mitotic cells and cell area, based on their direct interpretability and to facilitate comparability of our data with those of [1]. Subsequent features were selected by the following criterion. For each candidate feature m, we fit a linear regression that modeled this feature's values over all experiments as a function of the already selected features,

$$Y_{\cdot\cdot\cdot lm} \sim Y_{\cdot\cdot\cdot lM} \tag{4}$$

where M denotes the set of already selected features and $\sim$ indicates that a model is being fit that aims to predict the left hand side from the values on the right hand side. Let $\mathbf{r}_m^l$ denote the residuals of this fit. The magnitude of $\mathbf{r}_m^l$ is a measure of unique information content in feature m that is not yet covered by the already selected features M. This unique information content is a combination of two components: measurement noise and systematic signal. To decompose this combination, we computed the correlation coefficient between $\mathbf{r}_m^1$ and $\mathbf{r}_m^2$. Among all available candidate features, we selected the one with the maximum correlation and added it to the set M. We repeated this procedure until all features were exhausted. As a result, we obtained a ranking of the features by the order in which they were selected. The following figure shows the correlation coefficients of the first 50 features ranked in this manner.

```
library("DmelSGI")
library("RColorBrewer")
library("hwriter")
library("xtable")

basedir = getBaseDir()
resultdir = file.path( basedir, "result", "FeatureSelection")
dir.create(resultdir, recursive = TRUE,showWarnings=FALSE)
```

```
data("stabilitySelection", package="DmelSGI")
```

```
par(mar=c(12,5,0.5,0.5))
barplot(stabilitySelection$correlation,
        names.arg=hrNames(stabilitySelection$selected),las=2,
        col=ifelse(stabilitySelection$ratioPositive > 0.5,
                   brewer.pal(3,"Pastel1")[2],
                   brewer.pal(3,"Pastel1")[1]),
        ylab = "correlation", cex.lab=1.3)
```

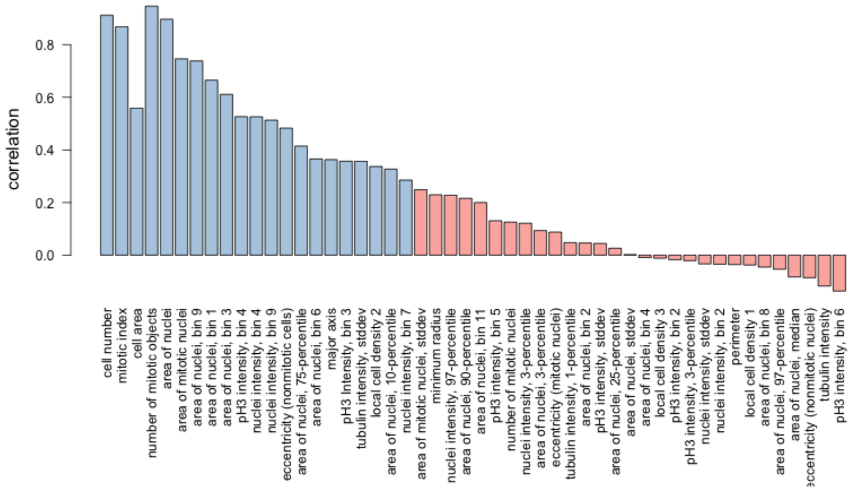

To determine the cutoff for the number of features to use in the subsequent analyses, we considered the distribution of correlation coefficients of the candidate features at each iteration. The following figure shows, for each iteration, the fraction of features with positive correlation coefficients.

```
par(mar=c(12,5,0.5,0.5))
barplot(stabilitySelection$ratioPositive-0.5,
        names.arg=hrNames(stabilitySelection$selected),las=2,
        col=ifelse(stabilitySelection$ratioPositive > 0.5,
                   brewer.pal(3,"Pastel1")[2],
                   brewer.pal(3,"Pastel1")[1]),
        ylab = "ratio positive cor.", cex.lab=1.3,
        offset=0.5)
```

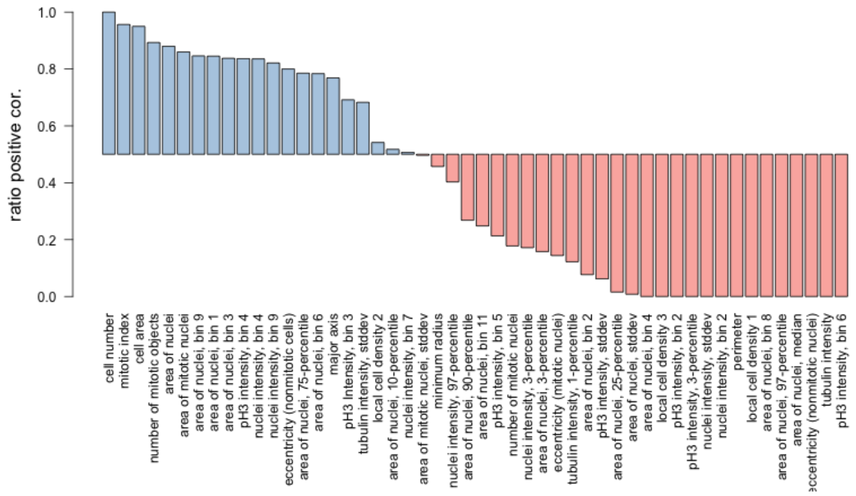

If the set of remaining candidate features is completely uninformative (i. e. if they only contain independent noise), then the distribution of their correlation coefficients is centered symmetrically around 0, and the fraction is expected to be $1/2$. Based on this observation, we defined as a cutoff criterion that the fraction of positive correlation coefficients should be larger than $1/2$, which led to the selection 21 features. These are marked in blue in the figure above and listed on the following table.

```
data("stabilitySelection", package="DmelSGI")
data("datamatrix", package="DmelSGI")
```

```
df = as.data.frame(stabilitySelection[c("selected","correlation","ratioPositive")],
                   stringsAsFactors=FALSE)
row.names(df) = 1:nrow(df)
df = cbind(df, Name=hrNames(stabilitySelection$selected),
           Selected = ifelse(stabilitySelection$ratioPositive > 0.5,"Selected",""),
           stringsAsFactors=FALSE)
df = df[,c(1,4,2,3,5)]
colnames(df) = c("ID","Name","Correlation","RatioPositive","Selected")
write.table(df, file=file.path(resultdir,"StabilitySelectedFeatures.txt"), sep="\t",
            quote=FALSE,row.names=FALSE)
```

The head of the table of selected features looks like this:

```
XT = xtable(df[1:7,],caption="List of features selected by stability")
label(XT) = "TabStabilitySelection"
print(XT,caption.placement="top")
```

The data remaining after feature selection were represented in a 5-dimensional array Y_{ijklm} with dimensions

- 1293 target genes,
- 2 dsRNA designs per target gene,
- 72 query genes,
- 2 dsRNA designs per query gene,
- 21 features.

Table 3: List of features selected by stability

	ID	Name	Correlation	RatioPositive	Selected
1	4x.count	cell number	0.91	1.00	Selected
2	4x.ratioMitotic	mitotic index	0.87	0.96	Selected
3	10x.meanNonmitotic.cell.0.s.area	cell area	0.56	0.95	Selected
4	4x.countpH3	number of mitotic objects	0.95	0.89	Selected
5	4x.areaNucAll	area of nuclei	0.90	0.88	Selected
6	4x.areapH3All	area of mitotic nuclei	0.75	0.86	Selected
7	4x.areaNucH9	area of nuclei, bin 9	0.74	0.85	Selected

8 Pairwise interaction scores

```
library("DmelSGI")
library("RColorBrewer")

basedir = getBaseDir()
resultdir = file.path( basedir, "result", "PairwiseInteractionScores")
dir.create(resultdir, recursive = TRUE,showWarnings=FALSE)

data("datamatrix", package="DmelSGI")
```

Pairwise interaction scores were computed from Y_{ijklm} for the 21 features selected in Section 7 by robust fit of a linear model. First, *main effects* were estimated by minimizing

$$(\hat{w}_m, \hat{\mu}_{ijm}, \hat{\nu}_{klm}) = \arg\min \sum_{ijkl} \|Y_{ijklm} - w_m - \mu_{ijm} - \nu_{klm}\|_1$$
$$\text{s.t.} \sum_{i \in \text{neg}} \mu_{ijm} = 0 \text{ and } \sum_{k \in \text{neg}} \nu_{klm} = 0 \quad (5)$$

Here, μ_{ijm} represents the effect on feature m of single knock down of dsRNA j for target gene i, ν_{klm} represents the effect on feature m of single knock down of dsRNA l for query gene k and w_m represents the baseline value of feature m. Separate estimates according to (5) were computed for each experimental batch, to let the parameters $\hat{w}_m$, $\hat{\mu}_{ijm}$, $\hat{\nu}_{klm}$ absorb small but detectable technical variation that was associated with the batches. The baseline effect w_m was estimated using the negative controls for query and target genes. Negative controls were available on each target plate as well as a query in each batch.

Pairwise interaction scores were computed by subtracting the prediction from the non-interaction model from the observed values:

$$\pi_{ijklm} = Y_{ijklm} - \hat{w}_m - \hat{\mu}_{ijm} - \hat{\nu}_{klm} \quad (6)$$

To summarize the four measurements for each gene pair (i, k), corresponding to the four dsRNA combinations of $j = 1, 2$ and $l = 1, 2$, the four values were tested against the null hypothesis that the interaction score is zero. p-values were computed by the moderated t-test implemented in the R package *limma*. The p-values were adjusted for multiple testing by the method of Benjamini-Hochberg [18]. A cut-off of 0.01 for the false discovery rate was chosen for all subsequent analyses that required the selection or reporting of pairwise genetic interactions.

8.1 Double knockdown examples

```
data("mainEffects",package="DmelSGI")
data("pimatrix",package="DmelSGI")
```

```
examples = data.frame(
  ph = c("area","mitoticIndex"),
  targetGenes = c("FBgn0014020","FBgn0033029"),
  queryGenes=c("FBgn0030276","FBgn0261456"),
  stringsAsFactors=FALSE)
```

```
Effects = array(0, dim=c(nrow(examples), 4, nrow(pimatrix$Anno$phenotype)))
for (i in seq_len(nrow(examples))) {
  I = match(examples$targetGenes[i], pimatrix$Anno$target$TID)
  J = match(examples$queryGenes[i],    pimatrix$Anno$query$TID)
  B = pimatrix$Anno$query$Batch[J]
  TP = pimatrix$Anno$target$TargetPlate[I]
  Effects[i,1,] = apply(mainEffects$target[I,,B,],2,
                        mean, na.rm=TRUE)
  Effects[i,2,] = apply(mainEffects$query[J,,TP,],  2,
                        mean, na.rm=TRUE)
  Effects[i,3,] = Effects[i,1,] + Effects[i,2,]
  Effects[i,4,] = apply(pimatrix$D[I,,J,,],3,mean) + Effects[i,3,]
}

for (i in seq_len(nrow(examples))) {
  tg = pimatrix$Anno$target$Symbol[match(examples$targetGenes[i],pimatrix$Anno$target$TID)]
  qg = pimatrix$Anno$query$Symbol[match(examples$queryGenes[i],
                                        pimatrix$Anno$query$TID)]
  pdf(file.path(resultdir,sprintf("ExamplePhenotypes-doubleKD-%s-%s.pdf",
              tg,qg)),height=8)
  par(mfrow=c(1,2),mar=c(18,3,3,0.2))
  K=1
  bp = barplot(Effects[i,,K],main="number cells",
               col=brewer.pal(3,"Pastel1")[1],cex.main=2,cex.axis=1.5)
  abline(h=0.0)
  lines(c(bp[4]-0.5,bp[4]+0.5),c(Effects[i,3,K],Effects[i,3,K]))
  arrows(x0=bp[4],Effects[i,3,K],x1=bp[4],Effects[i,4,K],code=3,
        length=min(0.25,abs(Effects[i,4,K] - Effects[i,3,K])))
  axis(side=1,at=bp,labels=c(sprintf("%s+ctrl.",tg),
                             sprintf("%s+ctrl.",qg),"expected",
                             sprintf("%s+%s",tg,qg)),col=NA,cex.axis=1.8,las=2)
  if (examples$ph[i] == "area") {
    K = 5
  } else {
    K = 2
  }
  bp = barplot(Effects[i,,K],main=ifelse(K==2,"mitotic index","nuclear area"),
               col=brewer.pal(3,"Pastel1")[2],cex.main=2,cex.axis=1.5)
  abline(h=0.0)
  lines(c(bp[4]-0.5,bp[4]+0.5),c(Effects[i,3,K],Effects[i,3,K]))
  arrows(x0=bp[4],Effects[i,3,K],x1=bp[4],Effects[i,4,K],code=3)
  axis(side=1,at=bp,labels=c(sprintf("%s + ctrl.",tg),
                             sprintf("%s + ctrl.",qg),"expected",
                             sprintf("%s + %s",tg,qg)),col=NA,
      cex.axis=1.8,las=2)
  dev.off()
}
```

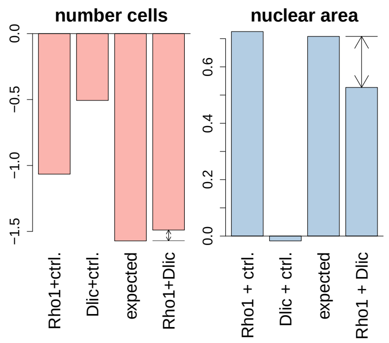

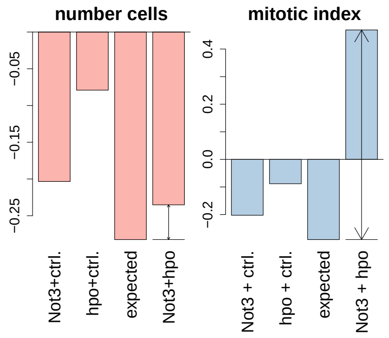


```r
data("Interactions", package="DmelSGI")

f = "4x.count"
names = c("mor","brm","Bap60","Snr1","osa")
```

```r
bp=barplot(Interactions$piscore[names, "RasGAP1", f],
           ylim=c(-0.6,0.6),ylab=sprintf("pi-score (%s)",hrNames(f)),
           las=2,cex.axis=1.5,cex.names=1.5,cex.lab=1.5,yaxp=c(-0.5,0.5,2))
abline(h=0)
```

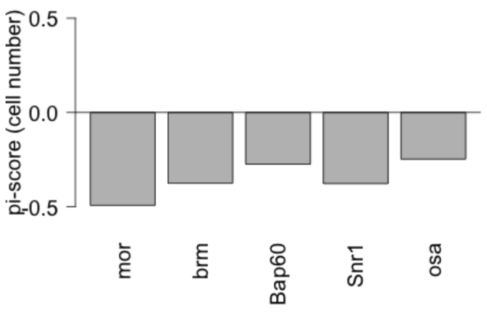

8.2 Main result table

```r
library("DmelSGI")

data("Interactions",package="DmelSGI")
```

```
PI = Interactions$piscore
PADJ = Interactions$padj
PI[is.na(PADJ)] = NA
```

The 3-dimensional arrays of π-scores and adjusted p-values are reshaped to a 2-dimensional matrix with one column per phenotypic feature.

```
dim(PI) = c(prod(dim(PI)[1:2]),dim(PI)[3])
dim(PADJ) = c(prod(dim(PADJ)[1:2]),dim(PADJ)[3])
```

The two matrices are merged in a way such that π-scores and adjusted p-values are interlaced and the two columns per features are next to each other.

```
V = cbind(PI, PADJ)
V = V[,rep(seq_len(dim(PI)[2]),each=2)+rep(c(0,dim(PI)[2]),times=dim(PI)[2])]
colnames(V) = sprintf("%s.%s",rep(c("pi-score","padj"),times=dim(PI)[2]),
        rep(hrNames(Interactions$Anno$phenotype$phenotype),
                          each=2))
```

Annotation of the target and query gene names are added to the table. The table is written to a text file.

```
target =       rep(Interactions$Anno$target$Symbol,
                   times=dim(Interactions$piscore)[2])
query =        rep(Interactions$Anno$query$Symbol,
                   each=dim(Interactions$piscore)[1])

df =  data.frame(targetGene=target,
                 queryGene=query,
                 V)

write.table(df, file=file.path(resultdir,"interactions.txt"),sep="\t",
            row.names=FALSE,quote=FALSE)
```

8.3 Comparison of main effects across different phenotypes

Average main effects (single knockdown effects) over dsRNA designs and batches.

```
data("mainEffects", package="DmelSGI")
D = apply(mainEffects$target, c(1,4), mean, na.rm=TRUE)
colnames(D) = hrNames(colnames(D))
```

Scatter plots of target main effects for the first 11 features selected by stability.

```
par(mar=c(0.2,0.2,0.2,0.2))
pairs(D[,1:11],pch=20,cex=0.5)
```

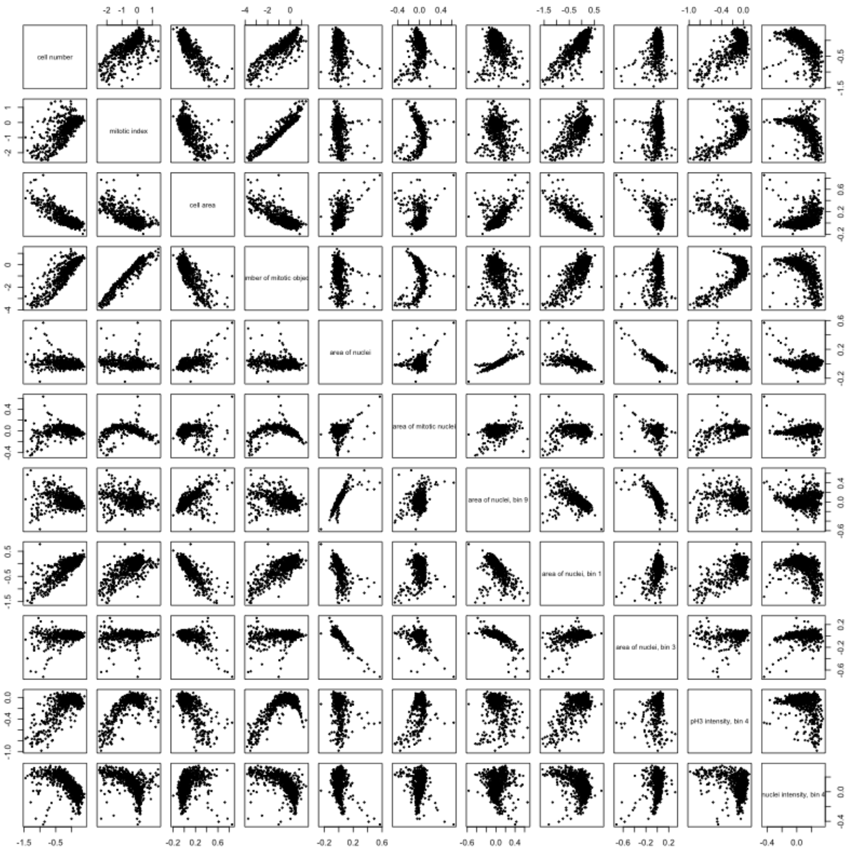

Scatter plots of target main effects for the features 12 to 21 selected by stability (and number of nuclei for comparison).

```
par(mar=c(0.2,0.2,0.2,0.2))
pairs(D[,c(1,12:21)],pch=20,cex=0.5)
```

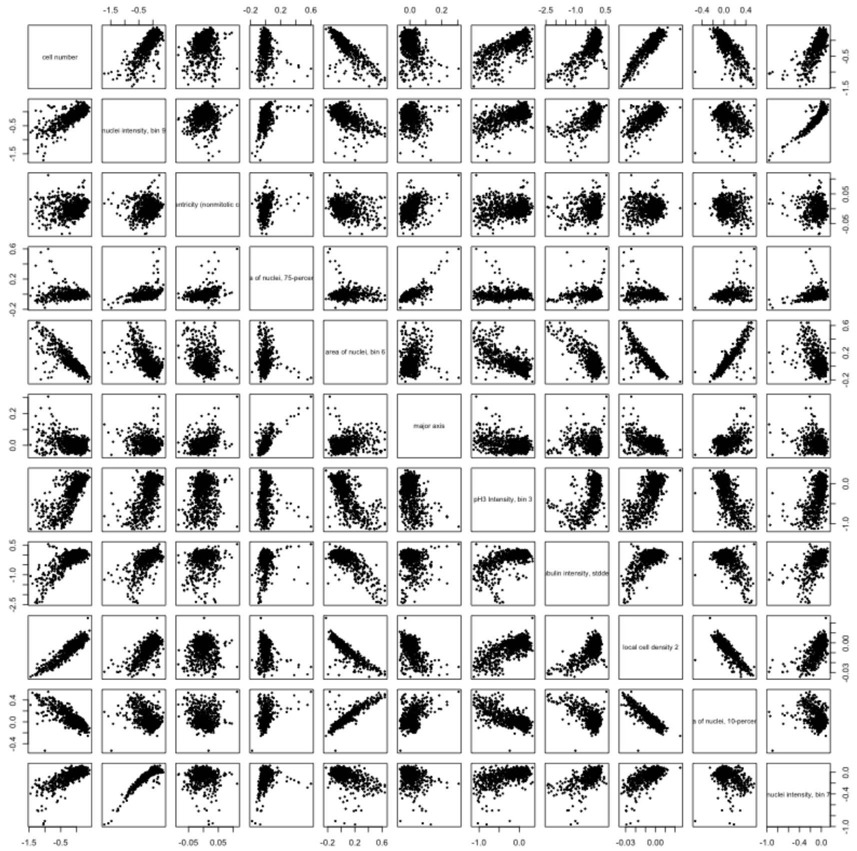

8.4 Comparison of main effects across batches

Average main effects of mitotic index (single knockdown effects) over dsRNA designs.

```
data("mainEffects", package="DmelSGI")
data("pimatrix", package="DmelSGI")
D = apply(mainEffects$target[,,,2], c(1,3), mean, na.rm=TRUE)
```

Scatter plots of mitotic index target main effects (number of nuclei) for the 12 batches.

```
par(mar=c(0.2,0.2,0.2,0.2))
pairs(D,pch=20,cex=0.5)
```

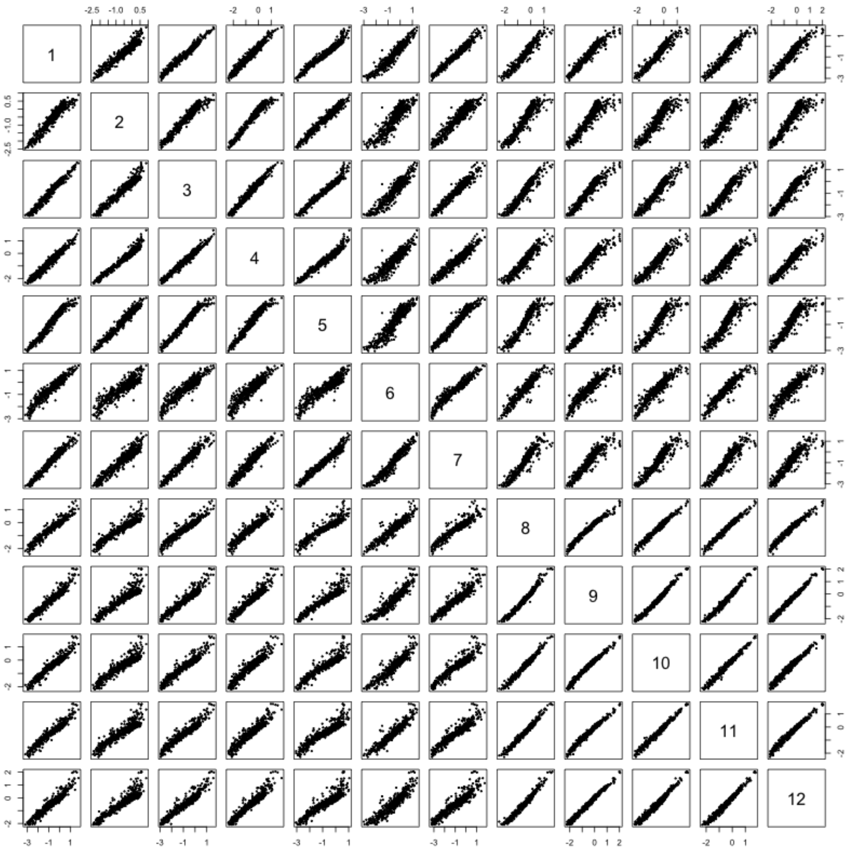

Scatter plots of mitotic index single knock down effects (number of nuclei) for batches one and twelve.

```
par(mar=c(4.5,4.5,1,1))
plot(D[,1],D[,12],pch=20,
     xlab="mitotic index [glog] in batch 1",
     ylab="mitotic index [glog] in batch 12",
     main="",cex=1.5,cex.lab=2,cex.axis=2,cex.main=2)
text(x=1,y=-2.0,sprintf("cor = %0.2f",cor(D[,3],D[,4])),cex=2)
```

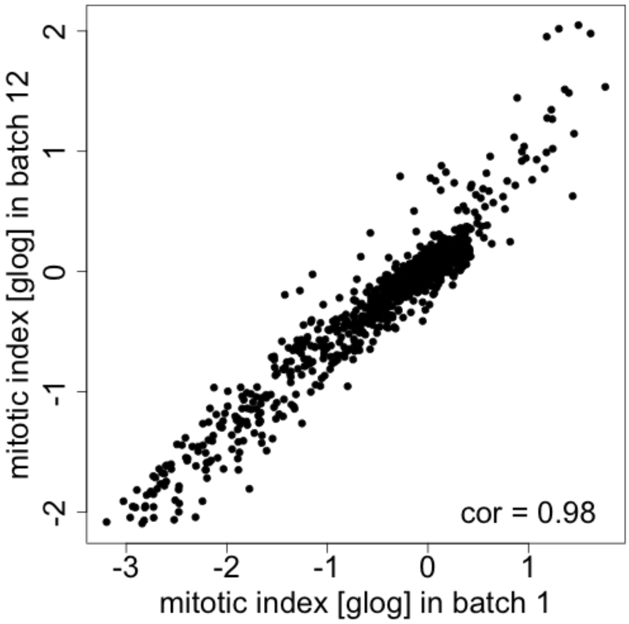

8.5 Comparison of pairwise interaction scores across different phenotypes

Load matrix of all pairwise interaction scores. Flatten dimension of genetic interaction cube along gene pairs.

```
data("Interactions", package="DmelSGI")
D = Interactions$piscore
dim(D) = c(prod(dim(D)[1:2]),dim(D)[3])
colnames(D) = hrNames(Interactions$Anno$phenotype$phenotype)
```

Sample gene pairs to avoid overplotting.

```
set.seed(1043289201)
S = sample(1:dim(D)[1],1000)
D1 = D[S,]
```

Scatter plots of pairwise interaction scores for the first 11 features selected by stability.

```
par(mar=c(0.2,0.2,0.2,0.2))
pairs(D1[,1:11],pch=20,cex=0.5)
```

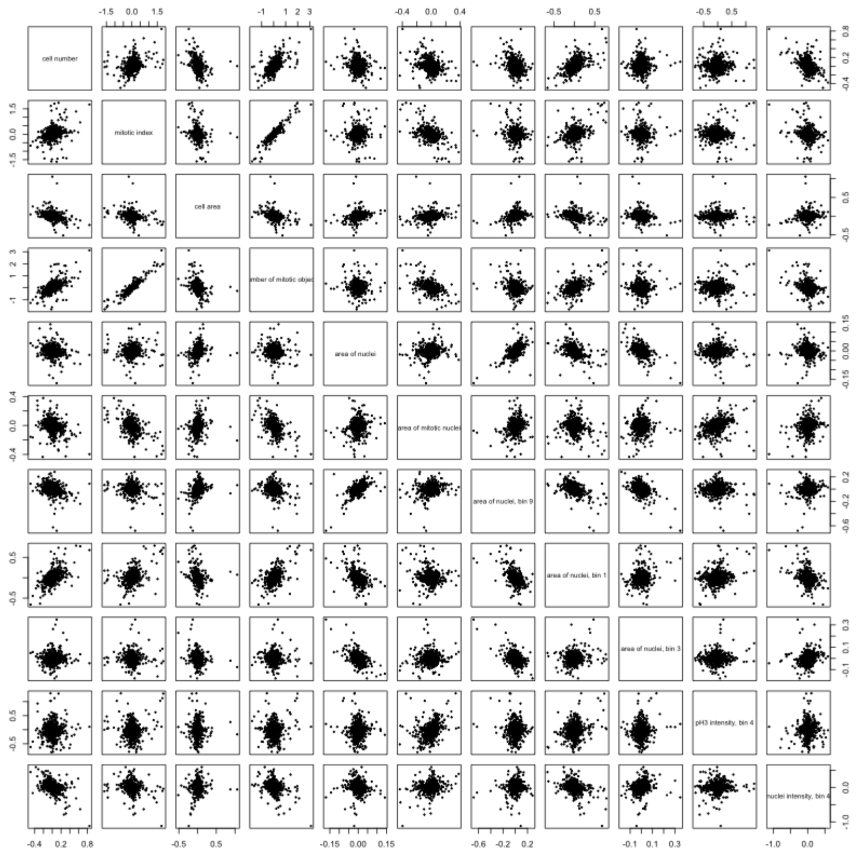

Scatter plots of pairwise interaction scores for the features 12 to 21 selected by stability (and number of nuclei for comparison).

```
par(mar=c(0.2,0.2,0.2,0.2))
pairs(D1[,c(1,12:21)],pch=20,cex=0.5)
```

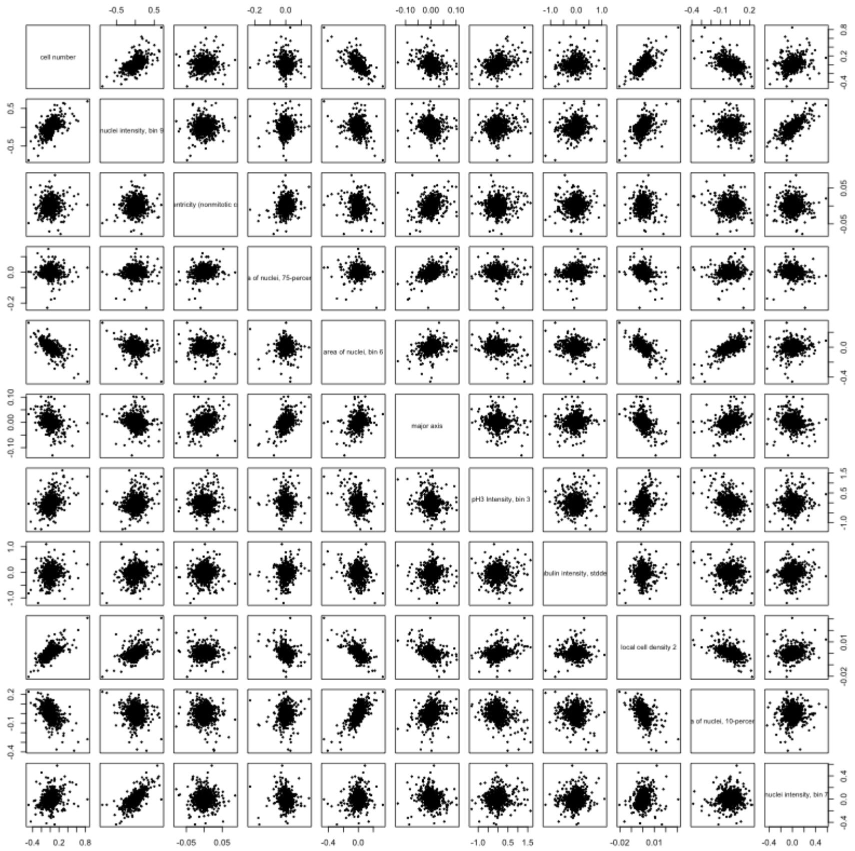

Scatter plots of pairwise interaction scores for nr of nuclei and mitotic ratio and major axis. Subsample gene pairs to avoid overplotting.

```
set.seed(1043289201)
S = sample(1:dim(D)[1],2000)
D1 = D[S,]
```

```
colnames(D1) = hrNames(colnames(D1))
for (i in c(2,16)) {
  X = D1[,c(1,i)]
  pdf(file=file.path(resultdir,sprintf("GeneticInteractionQC-piscore-cellnumber-%s.pdf",
                  gsub("[ ,()]","",colnames(X)[2]))))
  s = mad(X[,2],na.rm=TRUE) / mad(X[,1],na.rm=TRUE)
  r = max(abs(X[,1]),na.rm=TRUE)
  r = c(-r,r)
  X[which(X[,2] > s*r[2]),2] = s*r[2]
  X[which(X[,2] < s*r[1]),2] = s*r[1]
  par(mar=c(4.5,4.5,1,1))
  plot(X[,2],X[,1],pch=20,
       xlab=sprintf("pi-score (%s)",colnames(X)[2]),
       ylab=sprintf("pi-score (%s)",colnames(X)[1]),
       main="",cex=1.5,cex.lab=2,cex.axis=2,cex.main=2,xlim=s*r,ylim=r)
  dev.off()
  cat("correlation nrCells - ",dimnames(D1)[[2]][i]," = ",
      cor(X[,2],X[,1],use="pairwise.complete"),"\n")
}

## correlation nrCells -  mitotic index  =  0.3163803
## correlation nrCells -  major axis  =  -0.3209859
```

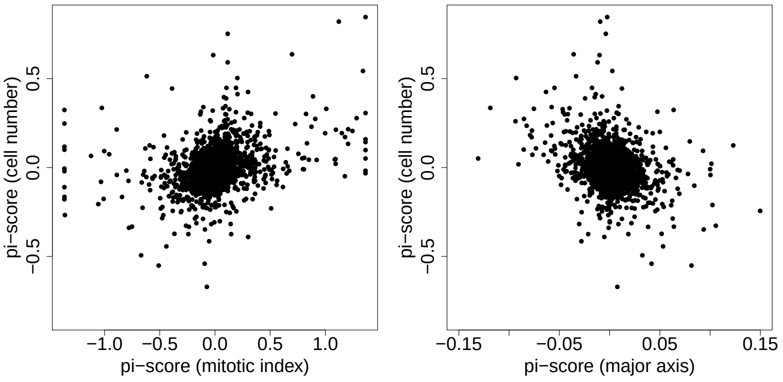

For comparison the correlation of π-scores across replicates is computed.

```
data("pimatrix", package="DmelSGI")
D = pimatrix$D
D1 = (D[,1,,1,] + D[,1,,2,]) / 2.0
D2 = (D[,2,,1,] + D[,2,,2,]) / 2.0
dim(D1) = c(prod(dim(D1)[1:2]),dim(D1)[3])
dim(D2) = c(prod(dim(D2)[1:2]),dim(D2)[3])
colnames(D1) = colnames(D2) = hrNames(pimatrix$Anno$phenotype$phenotype)

for (i in c(1,2,16)) {
  cc = cor(D1[,i],D2[,i],use="pairwise.complete")
  cat("correlation between replicates ",dimnames(D1)[[2]][i]," = ",cc,"\n")
}

## correlation between replicates  cell number  =  0.599544
## correlation between replicates  mitotic index  =  0.742961
## correlation between replicates  major axis  =  0.4390607
```

8.6 Numbers of genetic interactions

The number of positive and negative pairwise interactions per phenotype at a false discovery rate of 0.01 is enumerated.

```
data("Interactions", package="DmelSGI")
p.value.cutoff = 0.01
N = matrix(NA_integer_, nr=nrow(Interactions$Anno$phenotype), nc=2)
colnames(N) = c("pos","neg")
for (i in 1:nrow(Interactions$Anno$phenotype)) {
  PI = Interactions$piscore[,,i]
  N[i,2] = sum(PI[Interactions$padj[,,i] <= p.value.cutoff] > 0)
  N[i,1] = sum(PI[Interactions$padj[,,i] <= p.value.cutoff] < 0)
}
```

The number of interactions is converted in a fraction of interactions.

```
N = N / prod(dim(Interactions$piscore)[1:2])
```

Fraction of genetically interacting gene pairs (p-adj ¡= 0.01) (blue: negative, yellow positive) for each selected feature are plotted.

```
par(mar=c(15,5,0.5,0.5))
barplot(t(N),col=c("cornflowerblue","yellow"),
        names.arg=hrNames(Interactions$Anno$phenotype$phenotype),
        las=2, ylab ="fraction of interactions",
        cex.names=1.2,cex.axis=1.5,cex.lab=1.5)
```

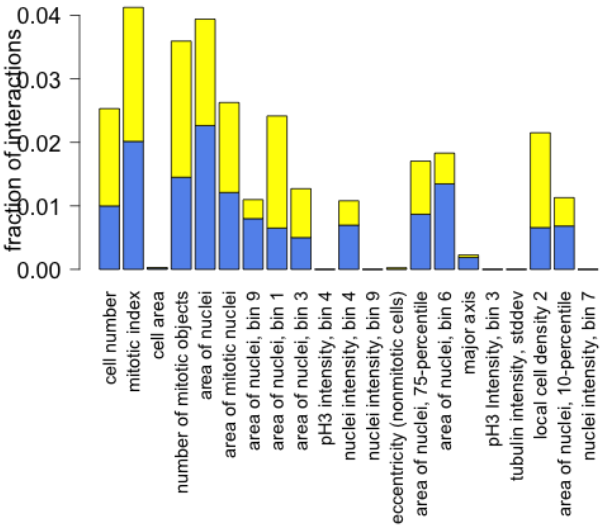

The cumulative fraction of interactions over the phenotypes is computed.

```
isinteraction = rep(FALSE, prod(dim(Interactions$piscore)[1:2]))
Ncum = rep(NA_integer_, nrow(Interactions$Anno$phenotype))
for (i in 1:nrow(Interactions$Anno$phenotype)) {
  isinteraction[Interactions$padj[,,i] <= p.value.cutoff] = TRUE
  Ncum[i] = sum(isinteraction) / prod(dim(Interactions$piscore)[1:2])
}
```

The cumulative fraction of genetically interacting gene pairs (p-adj ¡= 0.01) for the selected features are plotted.

```
par(mar=c(15,5,0.5,0.5),xpd=NA)
bp=barplot(Ncum, col=brewer.pal(3,"Pastel1")[2],
        ylab="fraction of interactions",las=2,
        names.arg = rep("",length(Ncum)),cex.axis=1.5,cex.lab=1.5)
text(bp,-0.01,hrNames(Interactions$Anno$phenotype$phenotype),adj=c(1,0.5),srt=38,cex=1.2)
```

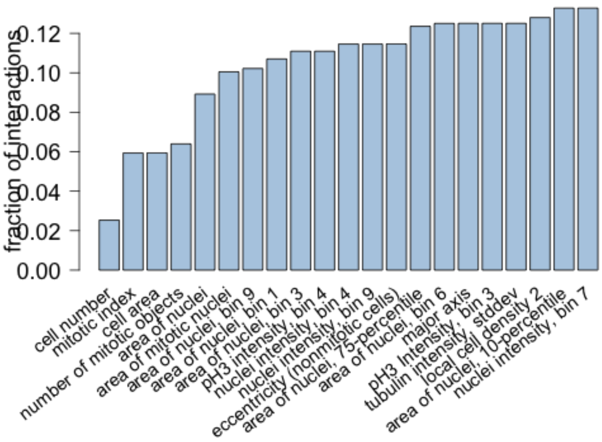

9 Multi-phenotype genetic interaction cube

```
library("DmelSGI")
library("grid")
library("RColorBrewer")
```

```
library("gplots")
library("beeswarm")

basedir = getBaseDir()
resultdir = file.path( basedir, "result", "GeneticInteractionCube")
dir.create(resultdir, recursive = TRUE,showWarnings=FALSE)

dir.create(file.path("Figures","GeneticInteractionCube"), showWarnings=FALSE)
```

9.1 Heatmap of genetic interaction cube

```
data("Interactions", package="DmelSGI")
```

π-scores are normalized through division by the median deviation to make them comparable across phenotypes. In this way π-scores are comparable to z-scores. *warning: Note: the following code chunk takes a long time to run. For convenience, the code is only shown but not run here, and its precomputed result is assigned in the subsequent code chunk.*

```
data("pimatrix", package="DmelSGI")
D = pimatrix$D
D2 = aperm(D, c(1,3,2,4,5))
dim(D2) = c(prod(dim(D2)[1:2]),prod(dim(D2)[3:4]),dim(D2)[5])

SD = apply(D2,c(1,3),sd, na.rm=TRUE)
MSD = apply(SD, 2, function(x) { median(x,na.rm=TRUE) } )
```

Hard coded result of the last code chunk.

```
MSD = c(0.0833528975114521, 0.134136618342975, 0.0498996012784751,
0.204772216536139, 0.0142975582945938, 0.0428299793772605, 0.0576235314621808,
0.0833934805305705, 0.0328437541652814, 0.147643254412127, 0.0866394118952878,
0.140840565863283, 0.0154131573539473, 0.0286467941877466, 0.0496616658001497,
0.0164694485385577, 0.233130597062897, 0.222961290060361, 0.00228512594775289,
0.0773453995034531, 0.0892678802977647)

D = Interactions$piscore
for (i in 1:dim(D)[3]) {
  D[,,i] = D[,,i] / MSD[i]
}
```

The π-scores are colorcoded showing negative interactions in blue and positive interactions in yellow. π-scores between -2 and 2 are colored black. The color range is capped at -6 and 6.

```
cuts = c(-Inf,
         seq(-6, -2, length.out=(length(DmelSGI:::colBY)-3)/2),
         0.0,
         seq(2, 6, length.out=(length(DmelSGI:::colBY)-3)/2),
         +Inf)
```

The three dimensions of the genetic interaction cube are ordered by hierarchical clustering.

```
ordTarget = orderDim(D, 1)
ordQuery = orderDim(D, 2)
ordFeat  = orderDim(D, 3)
Ph = c("4x.intNucH7","4x.count","4x.LCD2", "4x.ratioMitotic",
       "10x.meanNonmitotic.nucleus.DAPI.m.majoraxis", "4x.areaNucAll",
       "10x.meanNonmitotic.cell.Tub.m.eccentricity",
       "4x.areapH3All", "4x.intH3pH4")
D1 = D[ordTarget, ordQuery, Ph]
dimnames(D1)[[3]] = hrNames(dimnames(D1)[[3]])
```

The full heatmap of the three-dimensional genetic interaction cube is drawn (Blue: negative interactions, yellow: positive interactions).

9.2 Comparison to DPiM

The data is loaded.

```
library("DmelSGI")
library("RColorBrewer")
library("gplots")
library("grid")
data("Interactions",package="DmelSGI")
data("FBgn2anno",package="DmelSGI")
data("DPiM",package="DmelSGI")
```

The Pearson correlation coefficients between genetic interaction profiles are computed.

```
PI = Interactions$piscore
dim(PI) = c(dim(PI)[1],prod(dim(PI)[2:3]))
C = cor(t(PI))
row.names(C) = colnames(C) = Interactions$Anno$target$TID
```

Correlation coefficients are separated in two sets: gene pairs that are co-purified in the DPiM dataset [19] and gene pairs that are not co-purified in this dataset.

```
m1 = match(DPiM$interactions$Interactor_1, Interactions$Anno$target$TID)
m2 = match(DPiM$interactions$Interactor_2, Interactions$Anno$target$TID)
I = which(!is.na(m1) & !is.na(m2) & (m1 != m2))
I = cbind(m1[I],m2[I])
ccDPiM = C[I]
Iall = upper.tri(C)
Iall[I] = FALSE
Iall[cbind(I[,2],I[,1])] = FALSE
ccall = C[Iall]
```

The densities of correlation coefficients are plotted.

```
par(mar=c(5,5,0.2,5),xpd=NA)

d1 = density(ccDPiM, from=-1,to=1)
d2 = density(ccall, from=-1,to=1)

plot(d2, main="", col=brewer.pal(9, "Set1")[2],lwd=5,
     xlab="correlation of interaction profiles",ylim=c(0,1.5),
     cex.lab=1.8,cex.axis=1.8)
lines(d1$x,d1$y,col=brewer.pal(9, "Set1")[1],lwd=5)
legend("topleft",c("co-purified in DPiM","not captured by DPiM"),bty="n",
       fill = brewer.pal(9, "Set1")[1:2],cex=1.8)
```

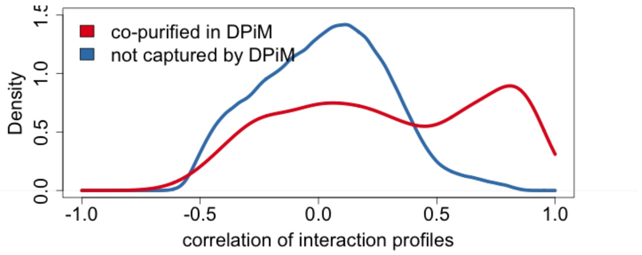

10 Genetic interaction landscape

```
library("DmelSGI")
library("igraph")
library("RSVGTipsDevice")
library("hwriter")
library("grid")

basedir = getBaseDir()
resultdir = file.path( basedir, "result", "GeneticInteractionLandscape")
dir.create(resultdir, recursive = TRUE,showWarnings=FALSE)
```

10.1 Correlation graph

```
data("Interactions", package="DmelSGI")
```

Preprocessing of the interaction data and correlation matrix

warning: Note: the following code chunk takes a long time to run. For convenience, the code is only shown but not run here, and its precomputed result is assigned in the subsequent code chunk. It computes normalization factors for each phenotypic feature. First, the standard deviation over the four siRNA combinations per gene pair per phenotype is computed. Then, the median of the standard deviations per phenotype is used as a normalization factor.

```
data("pimatrix", package="DmelSGI")
D = pimatrix$D
D2 = aperm(D, c(1,3,2,4,5))
dim(D2) = c(prod(dim(D2)[1:2]),prod(dim(D2)[3:4]),dim(D2)[5])

SD = apply(D2,c(1,3),sd, na.rm=TRUE)
MSD = apply(SD, 2, function(x) { median(x,na.rm=TRUE) } )
```

To avoid long waiting times, I hard coded the result of the last code chunk.

```
Sel = 1:1293
MSD = c(0.0833528975114521, 0.134136618342975, 0.0498996012784751,
0.204772216536139, 0.0142975582945938, 0.0428299793772605, 0.0576235314621808,
0.0833934805305705, 0.0328437541652814, 0.147643254412127, 0.0866394118952878,
0.140840565863283, 0.0154131573539473, 0.0286467941877466, 0.0496616658001497,
0.0164694485385577, 0.233130597062897, 0.222961290060361, 0.00228512594775289,
0.0773453995034531, 0.0892678802977647)
```

π- scores are devided by the median deviation computed in the last code chunk to make π-scores comparable across phenotypes. The genetic interaction profiles of the different phenotypes are stacked on top of each other.

```
PI = Interactions$piscore
for (i in 1:dim(PI)[3]) {
  PI[,,i] = PI[,,i] / MSD[i]
}
dim(PI) = c(dim(PI)[1],prod(dim(PI)[2:3]))
```

For each target gene the number of significant genetic interactions `nint` is computed. Then, for each gene pair i, j the minimum number of $\texttt{nint}_i$ and $\texttt{nint}_j$ is derived.

```
nint = apply(Interactions$padj <= 0.01,1,sum)
Nint = matrix(nint,nrow=length(nint),ncol=length(nint))
Nint[t(Nint) < Nint] = t(Nint)[t(Nint) < Nint]
row.names(Nint) = colnames(Nint) = Interactions$Anno$target$Symbol
```

Load the clusters that are highlighted in the correlation graph.

```
data("SelectedClusters", package="DmelSGI")
SelectedClusters = c(SelectedClusters,
      list(allOthers = Interactions$Anno$target$Symbol[
        !( Interactions$Anno$target$Symbol %in% unlist(SelectedClusters))]))
Labels = SelectedClusters
```

The genes are colored according to the clustering.

```
set.seed(26323)
Col = c("SWI/SNF" = "#ED1C24",
        "Condensin / Cohesin" = "#B8D433",
        "Cytokinesis" = "#67BF6B",
        "SAC" = "#3C58A8",
        "DREAM complex" = "#B64F9D",
        "Centrosome / Mitotic spindle" = "#EF4123",
        "CCT" = "#8CC63F",
        "Sequence-specific TFs" = "#67C4A4",
        "26S Proteasome" = "#3A51A3",
        "CSN" = "#F17A22",
        "RNA helicase" = "#6CBE45",
        "APC/C" = "#66C9D7",
        "Ribosomal biogenesis" = "#4A50A2",
        "Condensin / Cohesin (2)" = "#ED127A",
        "SAGA & Mediator" = "#F1B61C",
        "Cell-cell signalling" = "#61BB46",
        "Vesicle trafficking and cytoskeleton" = "#2CB4E8",
        "DNA repair and apoptosis" = "#6950A1",
        "ARP2/3 complex" = "#ED1940",
        "Tor signalling" = "#D54097",
        "Ras / MAPK signalling" = "#65BC46",
        "RNA PolII" = "#4074BA",
        "Wnt signalling" = "#8E4F9F")

col = rep("gray80",nrow(Interactions$Anno$target))
names(col) = Interactions$Anno$target$Symbol
for (i in 1:length(Labels)) {
  col[Labels[[i]]] = Col[i]
}
```

Perform a principal component analysis on the genetic interaction profiles and compute a correlation matrix after embedding of the genetic interaction profiles in 25 dimensions.

```
dimPCA = 25
PCA = prcomp(PI)
X = sweep(PI,2,PCA$center) %*% PCA$rotation[,1:dimPCA]
X = sweep(X,2,apply(X,2,sd), FUN="/")
theCorrPCA = cor(t(X), use = "pairwise.complete.obs")
row.names(theCorrPCA) = colnames(theCorrPCA) = Interactions$Anno$target$Symbol
```

The correlation matrix is converted in an Euclidean distance.

```
D = 2 - 2*theCorrPCA
D[lower.tri(D,diag=TRUE)] = NA
```

The Euclidean distance matrix is converted in an edge list. Edges with a distance larger than 0.8 (equates to a correlation smaller than 0.6) are discarded.

```
thresholdDist = 0.8
wedges = data.frame(V1 = rep(Interactions$Anno$target$Symbol,times=dim(D)[1]),
                    V2 = rep(Interactions$Anno$target$Symbol,each=dim(D)[1]),
                    nint = as.vector(Nint),
```

```
                    weight = as.vector(D),stringsAsFactors=FALSE)
wedges = wedges[which(wedges$weight <= thresholdDist),]
```

Create an *igraph*-object g.

```
g <- graph.data.frame(wedges, directed=FALSE)
V(g)$color = col[V(g)$name]
V(g)$frame.color = ifelse(V(g)$name %in% SelectedClusters$allOthers,
                          "#666666","#000000")
V(g)$size = 1.5
V(g)$size[!(V(g)$name %in% Labels$allOthers)] = 2.5
V(g)$label = rep("",length(V(g)$name))
E(g)$color <- "#e7e7e7"
E(g)$color[E(g)$nint > 5] <- "#cccccc"
```

The graph is layed-out by the Fruchterman-Reingold graph layout algorithm [20] using Euclidean distances subtracted from the maximum distance as edge weights.

```
set.seed(234816)
a = 0.07
b = 2.0
co <- layout.fruchterman.reingold(graph=g,
                          params=list(weights=(thresholdDist - E(g)$weight),
                          area=a*vcount(g)^2,
                          repulserad=b*a*vcount(g)^2*vcount(g)))
```

The gene coordinates are centered and scaled to a box in $[-1, 1]$.

```
co[,1] = 2*(co[,1] - min(co[,1])) / diff(range(co[,1]))-1
co[,2] = 2*(co[,2] - min(co[,2])) / diff(range(co[,2]))-1
row.names(co) = V(g)$name
```

The genes are permuted such that the colored vertices are drawn on top and the hairball is plotted.

```
g = permute.vertices(graph=g, permutation =
                       rank((!(V(g)$name %in% SelectedClusters$allOthers)),
                            ties.method="random"))
co = co[V(g)$name,]
```

```
# par(mar=c(0.1,0.1,0.1,0.1))
plot(g, layout=co)
plotHairballLabels(g, co, Labels[-length(Labels)], Col)
```

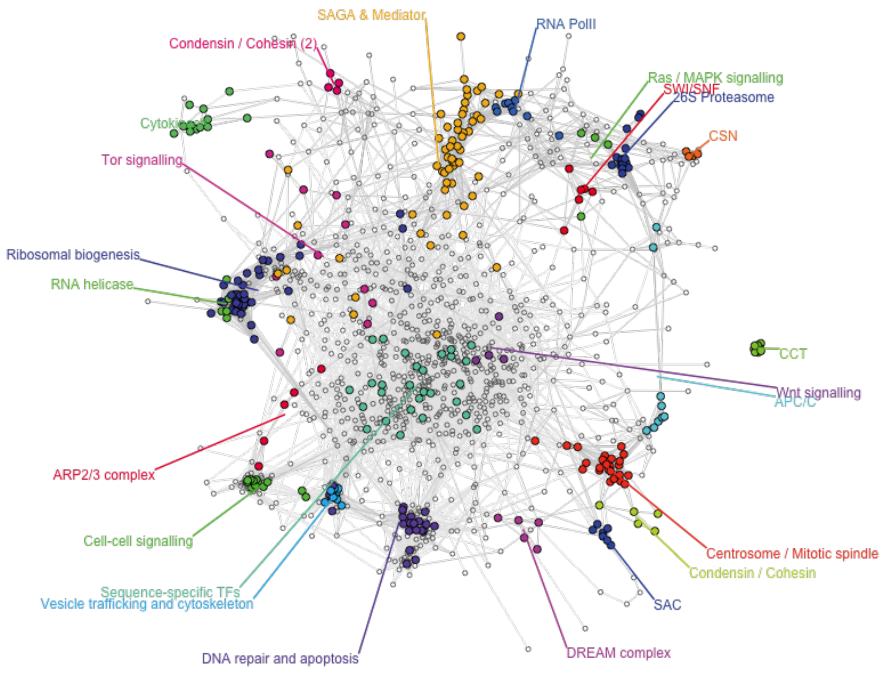

We zoom-in into some sub-graphs of the complete correlation graph. Therefore we select genes from mitosis, the translational machinary, and others that are highlighted in the paper.

The first sub-graph contains mitatic genes and shows the relation of the APC/C to the proteasome.

```
A =  SelectedClusters[c("APC/C","SAC","Centrosome / Mitotic spindle",
                        "Condensin / Cohesin","26S Proteasome")]
genesA = c("Arp10","fzy","vih","Klp61F","polo")
gsubA = induced.subgraph(g, which(V(g)$name %in% c(unlist(A),
                                                   genesA)))
```

Some clusters from the large hairball are subdivided in sub-clusters and obtain a new coloring. The sizes of the nodes and the gene names are increased.

```
data("SelectedClustersComplexes",package="DmelSGI")
V(gsubA)$color[which(V(gsubA)$name %in% SelectedClustersComplexes$gammaTuRC)] = "orange2"
V(gsubA)$color[which(V(gsubA)$name %in% SelectedClustersComplexes$'Dynein/Dynactin')] = "yellow2"
V(gsubA)$label = V(gsubA)$name
V(gsubA)$size = 8
V(gsubA)$label.cex = 0.4
V(gsubA)$label.color = "#222222"
E(gsubA)$color = "#777777"
```

The first sub-graph is plotted.

```
set.seed(38383)
LA = layout.fruchterman.reingold(gsubA,params=list(area=121^2*40))
plot(gsubA,layout=LA)
```

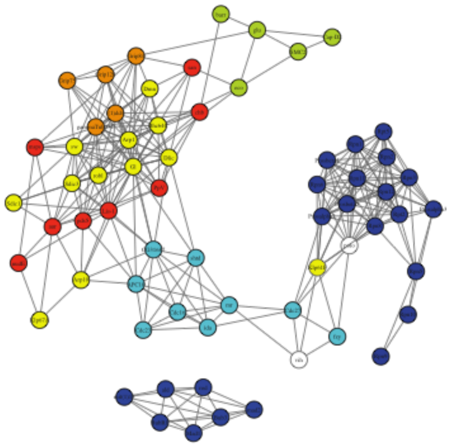

The second sub-graph contains ribosomal genes, signalling, and DNA repairs genes.

```
B =  SelectedClusters[c("Tor signalling","Ribosomal biogenesis","RNA helicase",
                        "Ribosomal biogenesis","RNA helicase",
                        "DNA repair and apoptosis","Cell-cell signalling",
                        "Vesicle trafficking and cytoskeleton")]
B$'Tor signalling' = B$'Tor signalling'[!(B$'Tor signalling' %in%
                                          c("trc","InR","gig","Tsc1","Pten"))]
genesB = c("Dbp45A","hpo","14-3-3epsilon",
           "CG32344", "CG9630", "kz", "pit", "Rs1","CG8545","twin")
gsubB = induced.subgraph(g, which(V(g)$name %in% c(unlist(B),
                                                  genesB)))
```

Some clusters from the large hairball are subdivided in sub-clusters and obtain a new coloring. The sizes of the nodes and the gene names are increased.

```
PolI = c("CG3756","l(2)37Cg","RpI1","RpI12","RpI135","Tif-IA")
PolIII = c("Sin","RpIII128","CG5380","CG12267","CG33051","CG7339")
V(gsubB)$color[which(V(gsubB)$name %in% PolI)] = "orange2"
V(gsubB)$color[which(V(gsubB)$name %in% PolIII)] = "yellow2"

V(gsubB)$label = V(gsubB)$name
V(gsubB)$size = 8
V(gsubB)$label.cex = 0.4
V(gsubB)$label.color = "#222222"
E(gsubB)$color = "#777777"
```

The second sub-graph is plotted.

```
set.seed(122138)
LB = layout.fruchterman.reingold(gsubB,params=list(area=121^2*40))
plot(gsubB,layout=LB)
```

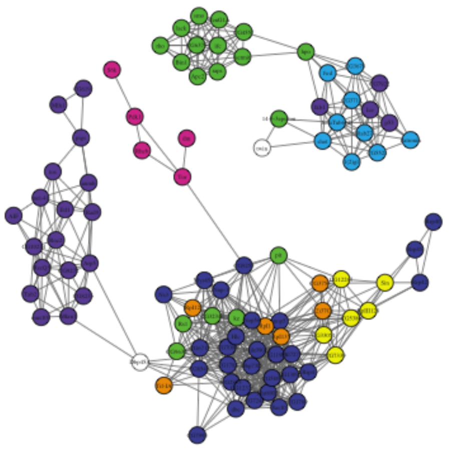

11 Directional epistatic interactions

```
library("DmelSGI")
library("abind")
library("igraph")

basedir = getBaseDir()
resultdir = file.path( basedir, "result", "DirectionalInteractions")
dir.create(resultdir, recursive = TRUE,showWarnings=FALSE)

data("Interactions", package="DmelSGI")
data("pimatrix", package="DmelSGI")
data("mainEffects", package="DmelSGI")
data("SelectedClustersComplexes", package="DmelSGI")
```

11.1 Inference of a directional, epistatic interactions

Extracting relevant π-scores and main effects.

```
pi  = pimatrix$D
mt  = mainEffects$target
mq  = mainEffects$query
```

Standardize the features by their 1%-99% percentiles.

```
myrange = function(x) diff(quantile(x, probs=c(0.01, 0.99), na.rm=TRUE))
featureSD = apply(pi, 5, myrange)
for(k in seq(along=featureSD)){
  pi[,,,,k] = pi[,,,,k]/featureSD[k]
  mt[,,,k]  = mt[,,,k]/featureSD[k]
  mq[,,,k]  = mq[,,,k]/featureSD[k]
}
```

Nested loops over target genes, target dsRNA designs, query genes, and query dsRNA designs. For each reagent pair, the π-score vector over all 21 phenotypes is fitted by a linear model as a function of the target main effects

and query main effects. The analysis of variance (anova) reports the fraction of variance that is described by the two main effects.

```
data = array(NA_real_, dim=c(21, 3, dim(pi)[1:4]))
for(it in seq_len(dim(pi)[1])) {
  targetplate = pimatrix$Anno$target$TargetPlate[ it ]
  for(dt in seq_len(dim(pi)[2])) {
    for(iq in seq_len(dim(pi)[3])) {
      batch = pimatrix$Anno$query$Batch[iq]
      xt = mt[it, dt, batch, ]
      for(dq in seq_len(dim(pi)[4])) {
        y    = pi[it, dt, iq, dq, ]
        xq   = mq[iq, dq, targetplate, ]
        nay  = sum(is.na(y))
        naxq = sum(is.na(xq))
        if((nay>1)||(naxq>1)) {
        } else {
          data[, 1, it, dt, iq, dq] = xt
          data[, 2, it, dt, iq, dq] = xq
          data[, 3, it, dt, iq, dq] = y
        } # else
      }
    }
  }
}

dimnames(data) = list(pimatrix$Anno$phenotype$phenotype,
                      c("xt","xq","pi"),
                      pimatrix$Anno$target$Symbol,
                      1:2,
                      pimatrix$Anno$query$Symbol,
                      1:2)
```

```
resCoef = array(NA_real_, dim=c(3, dim(pi)[1:4]))
resSq = array(NA_real_, dim=c(3, dim(pi)[1:4]))
resPV = array(NA_real_, dim=c(2, dim(pi)[1:4]))
for(it in seq_len(dim(pi)[1])) {
  for(dt in seq_len(dim(pi)[2])) {
    for(iq in seq_len(dim(pi)[3])) {
      for(dq in seq_len(dim(pi)[4])) {
        if (all(is.finite(data[, , it, dt, iq, dq]))) {
          model = lm(data[,3,it,dt,iq,dq] ~
                       data[,1,it,dt,iq,dq]+data[,2,it,dt,iq,dq])
          a = anova(model)
          resCoef[, it, dt, iq, dq] = model$coefficients
          resSq[1, it, dt, iq, dq] = a[1,2]
          resSq[2, it, dt, iq, dq] = a[2,2]
          resSq[3, it, dt, iq, dq] = a[3,2]
          resPV[1, it, dt, iq, dq] = a[1,5]
          resPV[2, it, dt, iq, dq] = a[2,5]
        } # else
      }
    }
  }
}

dimnames(resCoef) = list(c("const","xt","xq"),
                   pimatrix$Anno$target$Symbol,
                   1:2,
```

```
                    pimatrix$Anno$query$Symbol,
                    1:2)

dimnames(resSq) = list(c("xt","xq","res"),
                          pimatrix$Anno$target$Symbol,
                          1:2,
                          pimatrix$Anno$query$Symbol,
                          1:2)

dimnames(resPV) = list(c("xt","xq"),
                          pimatrix$Anno$target$Symbol,
                          1:2,
                          pimatrix$Anno$query$Symbol,
                          1:2)

fitepistasis = list(Coef = resCoef, Sq = resSq)
# save(fitepistasis, file="fitepistasis.rda")
```

The precomputed data from the last code chunk is loaded.

```
data("fitepistasis", package="DmelSGI")
```

The data for a directional epistatic interaction between Cdc23 and sti is shown for two phenotypes. Cdc23 is epistatic to sti.

```
plot2Phenotypes(data,"sti","Cdc23",1,5,lwd=3,cex.axis=1.3,cex.lab=1.3,cex=1.3)
```

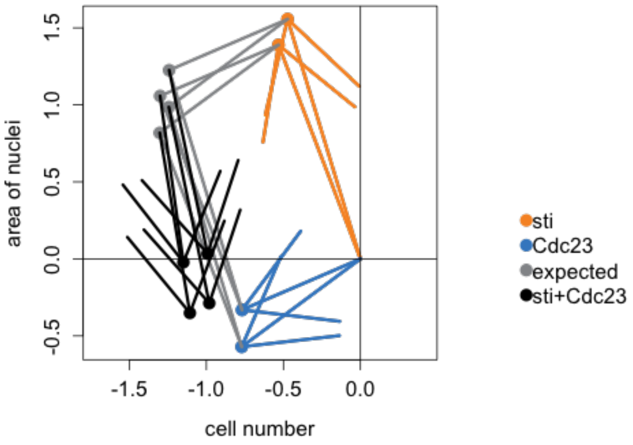

The data for a directional epistatic interaction between Cdc23 and sti is shown for all phenotypes.

```
plotPIdata(data,"sti","Cdc23",cex.axis=1.3,cex.lab=1.3)
```

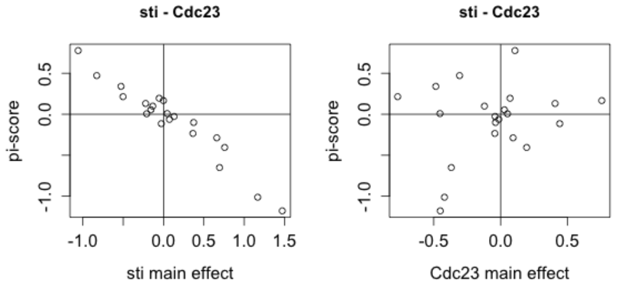

A directional epistatic network is derived from the linear model fits. The explained variance is converted in a fraction of explained variance.

```
resSq = fitepistasis$Sq
x = apply(resSq,2:5,sum, na.rm=TRUE)
resSq[1,,,,] = resSq[1,,,,] / x
resSq[2,,,,] = resSq[2,,,,] / x
resSq[3,,,,] = resSq[3,,,,] / x
```

Since target main effets show a better fit than query main effects, we applied different thresholds for target and query main effects to obtain equal chances of selection. For both, target and query main effects, we define a lower and a higher threshold as a 10% and 95% quantile.

```
NSIG = (apply(Interactions$padj <= 0.01,1:2,sum,na.rm=TRUE))
SQ = apply(resSq[,,,,], c(1,2,4),mean,na.rm=TRUE)
Coef = apply(fitepistasis$Coef[,,,,], c(1,2,4),mean,na.rm=TRUE)

thT = quantile(as.vector(SQ["xt",,]),probs=c(0.1,0.95))
thQ = quantile(as.vector(SQ["xq",,]),probs=c(0.1,0.95))
```

We report a directional epistatic interaction from target to query, if the target main effect explains less than 4.16and the query main effect explains more than 33.08

```
IT = which((NSIG >= 1) & (SQ["xt",,] > thT[2]) & (SQ["xq",,] < thQ[1]),arr.ind=TRUE)
IQ = which((NSIG >= 1) & (SQ["xq",,] > thQ[2]) & (SQ["xt",,] < thT[1]),arr.ind=TRUE)
IT = cbind(Interactions$Anno$target$Symbol[IT[,1]],
           Interactions$Anno$query$Symbol[IT[,2]])
IQ = cbind(Interactions$Anno$target$Symbol[IQ[,1]],
           Interactions$Anno$query$Symbol[IQ[,2]])

ET = data.frame(geneFrom = IT[,2], geneTo = IT[,1],
                sign = sign((Coef["xt",,])[IT]),
                coef = (Coef["xt",,])[IT],
                coefRev = NA,
                sqFrom = (SQ["xq",,])[IT],
                sqTo = (SQ["xt",,])[IT],
                mode = "target",
                stringsAsFactors=FALSE)
ITREV = cbind(IT[,2],IT[,1])
B = (ITREV[,1] %in% dimnames(Coef)[[2]]) &
  (ITREV[,2] %in% dimnames(Coef)[[3]])
ET$coefRev[B] = (Coef["xq",,])[ITREV[B,]]
EQ = data.frame(geneFrom = IQ[,1], geneTo = IQ[,2],
                sign = sign((Coef["xq",,])[IQ]),
                coef = (Coef["xq",,])[IQ],
                coefRev = NA,
                sqFrom = (SQ["xt",,])[IQ],
                sqTo = (SQ["xq",,])[IQ],
                mode = "query",
                stringsAsFactors=FALSE)
IQREV = cbind(IQ[,2],IQ[,1])
B = (IQREV[,1] %in% dimnames(Coef)[[2]]) &
  (IQREV[,2] %in% dimnames(Coef)[[3]])
EQ$coefRev[B] = (Coef["xt",,])[IQREV[B,]]
edges = rbind(ET,EQ)
edges$color = ifelse(edges$sign<0, "dodgerblue", "crimson")
```

We remove contradicting edges from the list.

```
key = sprintf("%s__%s",edges$geneFrom,edges$geneTo)
k = key[duplicated(key)]
g = tapply(edges$sign[key %in%  k],key[key %in%  k], function(x) {
  length(unique(x)) > 1})
```

```
if (any(g)) {
  cat(sum(key %in% names(which(g))),
      " edges with contraditing sign are removed.\n")
  edges = edges[!(key %in% names(which(g))),]
}
key = sprintf("%s__%s",edges$geneFrom,edges$geneTo)
edges = edges[!duplicated(key),]

key = sprintf("%s__%s",edges$geneFrom,edges$geneTo)
key2 = sprintf("%s__%s",edges$geneTo,edges$geneFrom)
if (any(key %in% key2)) {
  cat(sum(key %in% key2)," edges with contraditing direction are removed.\n")
  edges = edges[!((key %in% key2) | (key2 %in% key)),]
}

## 17  edges with contraditing direction are removed.
```

11.2 A table of all directional interactions

We identified in total 1344 directional epistatic interactions, thereof are 304 aggravating and 1040 alleviating interactions.

```
E = data.frame(edges[,c("geneFrom","geneTo")],
               effect=ifelse( edges$sign == 1, "aggravating", "alleviating"))
write.table(E, file=file.path(resultdir,"DirectionalEpistaticInteractions.txt"),
            sep="\t",row.names=FALSE,quote=FALSE)
```

11.3 An directional epistatic network for mitosis.

```
SelectedClustersComplexes =
  SelectedClustersComplexes[c("Cytokinesis","Condensin/Cohesin",
                              "SAC","Apc/C","gammaTuRC","Dynein/Dynactin")]
SelectedClustersComplexes$'Dynein/Dynactin' = c(SelectedClustersComplexes$'Dynein/Dynactin',
                                                "Klp61F")
SelectedClustersComplexes$polo = "polo"
SelectedClustersComplexes[["vih"]] = "vih"
SelectedClustersComplexes[["Elongin-B"]] = "Elongin-B"
SelectedClustersComplexes[["Skp2"]] = "Skp2"

QG = unlist(SelectedClustersComplexes)
edges2 = edges
edges2 = edges2[(edges2$geneFrom %in% QG) & (edges2$geneTo %in% QG),]
nodes2 = list()
for (cl in names(SelectedClustersComplexes)) {
  nodes2[[cl]] = unique(c(edges2$geneFrom, edges2$geneTo))
  nodes2[[cl]] = nodes2[[cl]][nodes2[[cl]] %in%
                                  SelectedClustersComplexes[[cl]] ]
}
cat(sprintf("Writing graph with %d nodes and %d edges.\n",
            length(nodes2), nrow(edges2)))

## Writing graph with 10 nodes and 63 edges.

out = c("digraph DirectionalInteractions {",
        paste("graph [size=\"10,10\" ratio=0.35 mode=major outputorder=edgesfirst overlap=false",
        "rankdir = \"LR\"];",sep=" "),
        "graph [splines=true];")
```

Some dummy nodes are inserted to better separate the complexes.

```
de = data.frame(
  from=c("CG31687","xxx","CG31687","xxx","Gl","xxx4","xxx4","rod","abcd1","polo","Grip75",
         "xxx2","Bub3","xxx3","Grip84","feo","xxx5","Apc10"),
  to  =c("xxx","cenB1A","xxx","Elongin-B","xxx4","Cdc23","vih","polo","polo","feo","xxx2",
         "Bub3","xxx3","Klp61F","polo","xxx5","glu","sti"),
  stringsAsFactors=FALSE)
for (i in seq_len(nrow(de))) {
  out <- c(out, rep(sprintf("\"%s\" -> \"%s\" [style=invis];",de$from[i],de$to[i]),5))
}
dg = c(de$from,de$to)
dg = unique(dg[!(dg %in% Interactions$Anno$target$Symbol)])
out <- c(out, sprintf("%s [style=invis]\n",dg))
```

```
  out <- c(out,
#            "newrank=true;",
#            "1 -> 2 -> 3 -> 4 -> 5 -> 6 -> 7 -> 8 -> 9 [style=invis];",
        sprintf("\"%s\" -> \"%s\" [color=\"%s\" penwidth=3 arrowsize=2];",
                edges2$geneFrom, edges2$geneTo, edges2$color))
for (cl in seq_along(SelectedClustersComplexes)) {
  out = c(out,sprintf("subgraph cluster%s {",LETTERS[cl]))
  out = c(out,
          sprintf(paste("\"%s\" [label=\"%s\" shape=ellipse style=filled fillcolor=\"%s\"",
                  "labelfontsize=%d margin=\"0.02,0.02\" tooltip=\"%s\"];"),
                  nodes2[[cl]], nodes2[[cl]],
                  "#F5ECE5",
                  20, nodes2[[cl]]))
  out = c(out, sprintf("label=\"%s\";",names(SelectedClustersComplexes)[cl]))
  out = c(out," }")
}
out = c(out,"}")

file = file.path(resultdir,"DirectionalInteractions-Mitosis")
writeLines(out, con=sprintf("%s.dot",file) )
```

If you have installed graphviz on your computer, you can now run dot to generate the figure.

```
system(sprintf("dot %s.dot -Tpdf -o%s.pdf",file,file))
```

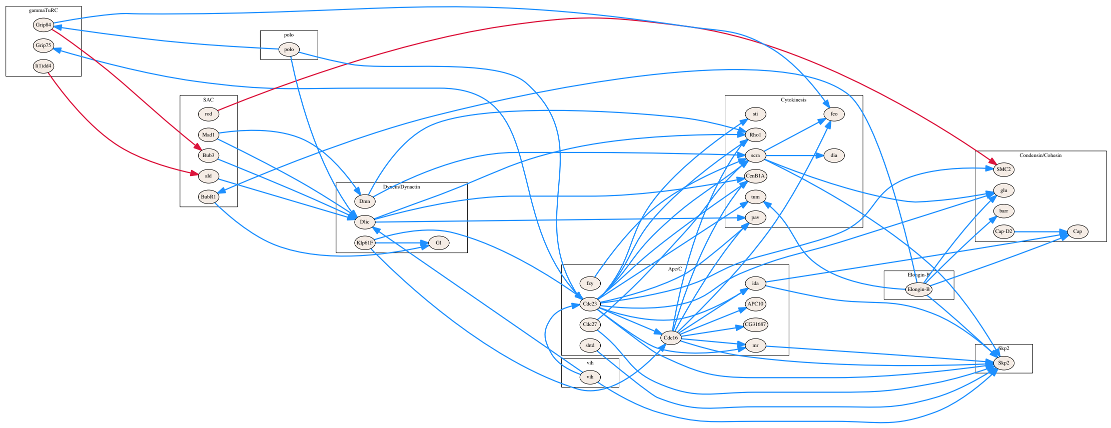


```
plot2Phenotypes(data,"polo","Grip84",2,3,lwd=1,cex.axis=0.5,cex.lab=0.5,cex=0.5,length = 0.1)
plot2Phenotypes(data,"BubR1","Elongin-B",5,19,lwd=1,cex.axis=0.5,cex.lab=0.5,cex=0.5,length = 0.1)
plot2Phenotypes(data,"rod","SMC2",1,4,lwd=1,cex.axis=0.5,cex.lab=0.5,cex=0.5,length = 0.1)
```

```
plot2Phenotypes(data,"Cdc23","SMC2",1,4,lwd=1,cex.axis=0.5,cex.lab=0.5,cex=0.5,length = 0.1)
plot2Phenotypes(data,"Klp61F","Cdc16",1,2,lwd=1,cex.axis=0.5,cex.lab=0.5,cex=0.5,length = 0.1)
plot2Phenotypes(data,"ald","l(1)dd4",4,19,lwd=1,cex.axis=0.5,cex.lab=0.5,cex=0.5,length = 0.1)
plot2Phenotypes(data,"ald","Dlic",2,19,lwd=1,cex.axis=0.5,cex.lab=0.5,cex=0.5,length = 0.1)
```

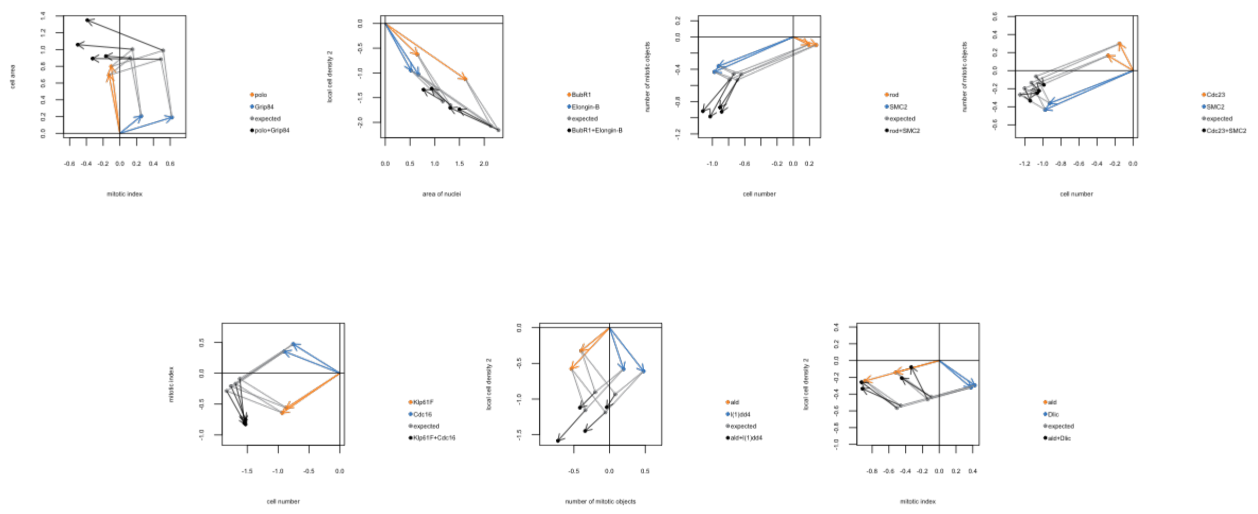

11.4 Subgraphs of the directional network

Draw some small examples of directional epistatic networks.

```
Genes = list("sti"=c("sti","Cdc23"),
             "RasGAP1" = c("RasGAP1", "dalao", "Snr1", "osa","brm", "mor",
                           "Bap60", "Dsor1", "Pvr", "Sos", "pnt"))

for (g in seq_along(Genes)) {
  QG = Genes[[g]]

  edges2 = edges
  edges2 = edges2[(edges2$geneFrom %in% QG)  & (edges2$geneTo %in% QG),]
  cat(sprintf("Writing graph with %d nodes and %d edges.\n", length(nodes2), nrow(edges2)))
  edges2$color[edges2$color == "crimson"] = "#DB1D3D"
  edges2$color[edges2$color == "dodgerblue"] = "#4C86C6"
  edges2$width = 5
  edges2$arrow.size = 2

  genes = data.frame(gene = unique(c(edges2$geneFrom,edges2$geneTo)),stringsAsFactors=FALSE)
  genes$color = rep("#F5ECE5",nrow(genes))
  genes$frame.color = NA
  genes$label.color = "black"
  genes$label.cex=1.3
  genes$size=50
  g = graph.data.frame(edges2,vertices = genes)

  set.seed(3122)
  plot(g)
}
```

```
plot2Phenotypes(data,"Snr1","RasGAP1",1,5,lwd=2,cex.axis=1,cex.lab=1,cex=1,length = 0.2)
plot2Phenotypes(data,"Snr1","RasGAP1",1,13,lwd=2,cex.axis=1,cex.lab=1,cex=1,length = 0.2)
plot2Phenotypes(data,"brm","RasGAP1",1,5,lwd=2,cex.axis=1,cex.lab=1,cex=1,length = 0.2)
plot2Phenotypes(data,"brm","RasGAP1",1,13,lwd=2,cex.axis=1,cex.lab=1,cex=1,length = 0.2)
plot2Phenotypes(data,"mor","RasGAP1",1,5,lwd=2,cex.axis=1,cex.lab=1,cex=1,length = 0.2)
plot2Phenotypes(data,"mor","RasGAP1",1,13,lwd=2,cex.axis=1,cex.lab=1,cex=1,length = 0.2)
```

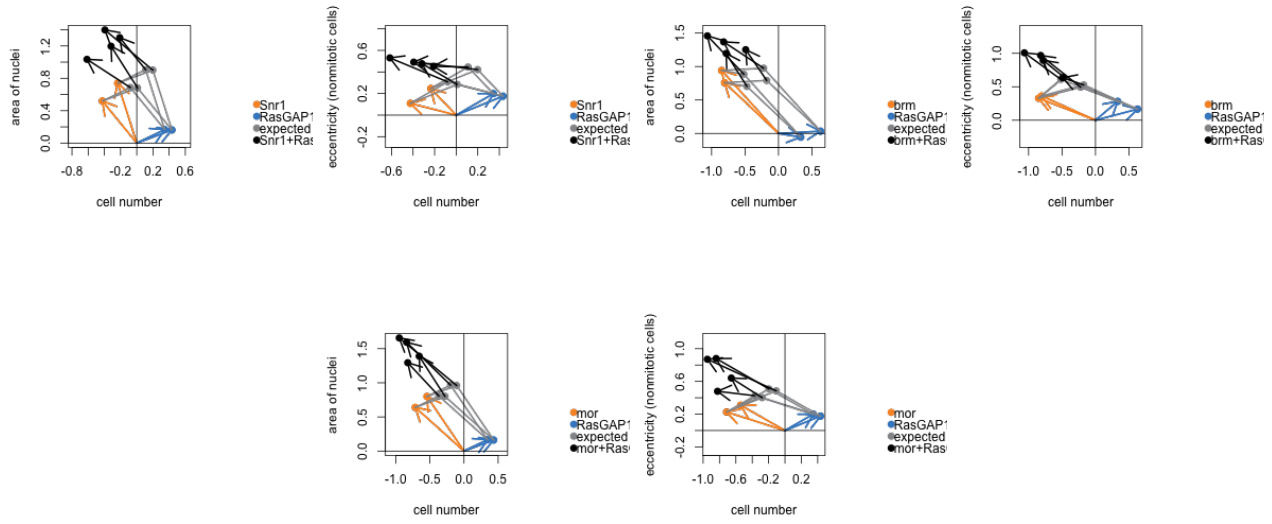

11.5 Directional interactions of recurrently mutated genes

```
data("TID2HUGO", package="DmelSGI")
data("Interactions", package="DmelSGI")
HugoNames = sapply(TID2HUGO, function(x) {
  if(length(x) > 0) {
    j=0
    for (i in 1:nchar(x[1])) {
      if (length(unique(substr(x,1,i))) == 1) {
        j=i
      }
    }
    res = paste(substr(x,1,j)[1],paste(substr(x,j+1,nchar(x)),collapse="/"),sep="")
  } else {
    res = ""
  }
  res
})
HugoNames = paste(Interactions$Anno$target$Symbol," (",HugoNames,")",sep="")
names(HugoNames) = Interactions$Anno$target$Symbol


data("Intogen", package="DmelSGI")

SelCancer = sapply(TID2HUGO, function(x) { any(x %in% Intogen$symbol) })
SelCancer = Interactions$Anno$target$Symbol[which(Interactions$Anno$target$TID %in%
                                                     names(which(SelCancer)))]

Genes = list("Pten" = c("Pten","gig"),
             "Arp3" = c("Arp3","Sos"),
```

```
              "Myb" = c("Myb","mip120","mip130","polo","fzy","Elongin-B","CtBP",
                        "sti","pav","tum","feo","Rho1","dia","scra","SMC4"),
              "nonC" = c("nonC","spen"),
              "Nup75" = c("Nup75","Sin3A","CtBP","jumu","RecQ4"))

for (g in seq_along(Genes)) {
  QG = Genes[[g]]

  edges2 = edges
  edges2 = edges2[(edges2$geneFrom %in% QG)  & (edges2$geneTo %in% QG),]
  edges2 = edges2[(edges2$geneFrom %in% QG[1])  | (edges2$geneTo %in% QG[1]),]
  cat(sprintf("Writing graph with %d nodes and %d edges.\n", length(nodes2), nrow(edges2)))
  edges2$color[edges2$color == "crimson"] = "#DB1D3D"
  edges2$color[edges2$color == "dodgerblue"] = "#4C86C6"
  edges2$width = 5
  edges2$arrow.size = 2

  genes = data.frame(gene = unique(c(edges2$geneFrom,edges2$geneTo)),stringsAsFactors=FALSE)
  genes$color = rep("#DDDDDC",nrow(genes))
  genes$color[genes$gene %in% SelCancer] = "#777777"
  genes$frame.color = NA
  genes$label.color = "black"
  genes$label.cex=1.5
  genes$size=50
  g = graph.data.frame(edges2,vertices = genes)
  V(g)$name = HugoNames[V(g)$name]

  set.seed(3122)
  plot(g)
  legend("bottomright", inset = c(-0.07,-0.07),fill=c("#777777", "#DDDDDC"),
         c("genes recurrently mutated in cancer","not recurrently mutated"),cex=0.5)
}
```

12 Session info

Here is the output of sessionInfo on the system on which this document was compiled:

```
sessionInfo()

## R version 3.1.2 (2014-10-31)
## Platform: x86_64-apple-darwin14.0.0/x86_64 (64-bit)
##
## locale:
## [1] de_DE.UTF-8/de_DE.UTF-8/de_DE.UTF-8/C/de_DE.UTF-8/de_DE.UTF-8
##
## attached base packages:
## [1] parallel  grid      stats     graphics  grDevices utils     datasets  methods
## [9] base
##
## other attached packages:
##  [1] RSVGTipsDevice_1.0-4  igraph_0.7.1          gplots_2.16.0
##  [4] xtable_1.7-4          hwriter_1.3.2         beeswarm_0.1.6
##  [7] RColorBrewer_1.1-2    RNAinteractMAPK_1.3.1 RNAinteract_1.14.0
## [10] Biobase_2.26.0        BiocGenerics_0.12.1   locfit_1.5-9.1
## [13] abind_1.4-0           sparseLDA_0.1-6       mda_0.4-4
## [16] class_7.3-12          MASS_7.3-39           elasticnet_1.1
## [19] lars_1.2              fields_8.2-1          maps_2.3-9
```

```
## [22] spam_1.0-1           DmelSGI_0.9.12       knitr_1.9
##
## loaded via a namespace (and not attached):
##  [1] affy_1.44.0           affyio_1.34.0         annotate_1.44.0
##  [4] AnnotationDbi_1.28.1  BiocInstaller_1.16.2  BiocStyle_1.4.1
##  [7] bitops_1.0-6          Category_2.32.0       caTools_1.17.1
## [10] cellHTS2_2.30.0       cluster_2.0.1         codetools_0.2-11
## [13] DBI_0.3.1             DEoptimR_1.0-2        digest_0.6.8
## [16] evaluate_0.5.5        formatR_1.0           gdata_2.13.3
## [19] genefilter_1.48.1     geneplotter_1.44.0    GenomeInfoDb_1.2.4
## [22] graph_1.44.1          GSEABase_1.28.0       gtools_3.4.1
## [25] highr_0.4             ICS_1.2-4             ICSNP_1.0-9
## [28] IRanges_2.0.1         KernSmooth_2.23-14    lattice_0.20-30
## [31] latticeExtra_0.6-26   limma_3.22.6          Matrix_1.1-5
## [34] mvtnorm_1.0-2         pcaPP_1.9-60          prada_1.42.0
## [37] preprocessCore_1.28.0 RBGL_1.42.0           rhdf5_2.11.7
## [40] robustbase_0.92-3     rrcov_1.3-8           RSQLite_1.0.0
## [43] S4Vectors_0.4.0       splines_3.1.2         splots_1.32.0
## [46] stats4_3.1.2          stringr_0.6.2         survey_3.30-3
## [49] survival_2.38-1       tools_3.1.2           TSP_1.0-10
## [52] vsn_3.34.0            XML_3.98-1.1          zlibbioc_1.12.0
```

References

[1] T. Horn, T. Sandmann, B. Fischer, E. Axelsson, W. Huber, and M. Boutros. Mapping of signaling networks through synthetic genetic interaction analysis by RNAi. *Nature Methods*, 8(4):341–346, 2011.

[2] F. P. Casey, G. Cagney, N. J. Krogan, and D. C. Shields. Optimal stepwise experimental design for pairwise functional interaction studies. *Bioinformatics*, 24:2733–2739, 2008.

[3] T. Horn, T. Sandmann, and M. Boutros. Design and evaluation of genome-wide libraries for RNA interference screens. *Genome Biol.*, 11(6):R61, 2010.

[4] G. Pau, F. Fuchs, O. Sklyar, M. Boutros, and W. Huber. EBImage - an R package for image processing with applications to cellular phenotypes. *Bioinformatics*, 26(7):979–981, 2010.

[5] F. Fuchs, G. Pau, D. Kranz, O. Sklyar, C. Budjan, S. Steinbrink, T. Horn, A. Pedal, W. Huber, and M. Boutros. Clustering phenotype populations by genome-wide RNAi and multiparametric imaging. *Molecular Systems Biology*, 6(1), 2010.

[6] M. Held, M.H.A. Schmitz, B. Fischer, T. Walter, B. Neumann, M.H. Olma, M. Peter, J. Ellenberg, and D.W. Gerlich. CellCognition: time-resolved phenotype annotation in high-throughput live cell imaging. *Nature Methods*, 7(9):747–754, 2010.

[7] A.E. Carpenter, T.R. Jones, M.R. Lamprecht, C. Clarke, I.H. Kang, O. Friman, D.A. Guertin, J.H. Chang, R.A. Lindquist, J. Moffat, et al. CellProfiler: image analysis software for identifying and quantifying cell phenotypes. *Genome Biology*, 7(10):R100, 2006.

[8] T. Jones, A. Carpenter, and P. Golland. Voronoi-based segmentation of cells on image manifolds. *Computer Vision for Biomedical Image Applications*, pages 535–543, 2005.

[9] R.M. Haralick, K. Shanmugam, and I.H. Dinstein. Textural features for image classification. *Systems, Man and Cybernetics, IEEE Transactions on*, 3(6):610–621, 1973.

[10] R. Mani, R.P. St Onge, J.L. Hartman, G. Giaever, and F.P. Roth. Defining genetic interaction. *Proceedings of the National Academy of Sciences*, 105(9):3461, 2008.

[11] M. Schuldiner, S.R. Collins, N.J. Thompson, V. Denic, A. Bhamidipati, T. Punna, J. Ihmels, B. Andrews, C. Boone, J.F. Greenblatt, et al. Exploration of the function and organization of the yeast early secretory pathway through an epistatic miniarray profile. *Cell*, 123(3):507–519, 2005.

[12] M. Costanzo, A. Baryshnikova, J. Bellay, Y. Kim, E.D. Spear, C.S. Sevier, H. Ding, J.L.Y. Koh, K. Toufighi, S. Mostafavi, et al. The genetic landscape of a cell. *Science*, 327(5964):425, 2010.

[13] A. Baryshnikova, M. Costanzo, Y. Kim, H. Ding, J. Koh, K. Toufighi, J.Y. Youn, J. Ou, B.J. San Luis, S. Bandyopadhyay, et al. Quantitative analysis of fitness and genetic interactions in yeast on a genome scale. *Nature Methods*, 7(12):1017–1024, 2010.

[14] S. Bandyopadhyay, M. Mehta, D. Kuo, M.K. Sung, R. Chuang, E.J. Jaehnig, B. Bodenmiller, K. Licon, W. Copeland, M. Shales, et al. Rewiring of genetic networks in response to DNA damage. *Science*, 330(6009):1385, 2010.

[15] E. Axelsson, T. Sandmann, T. Horn, M. Boutros, W. Huber, and B. Fischer. Extracting quantitative genetic interaction phenotypes from matrix combinatorial RNAi. *BMC Bioinformatics*, 12(1):342, 2011.

[16] W. Huber, A. Von Heydebreck, H. Sültmann, A. Poustka, and M. Vingron. Variance stabilization applied to microarray data calibration and to the quantification of differential expression. *Bioinformatics*, 18(suppl 1):S96–S104, 2002.

[17] Jennifer L Rohn, David Sims, Tao Liu, Marina Fedorova, Frieder Schöck, Joseph Dopie, Maria K Vartiainen, Amy A Kiger, Norbert Perrimon, and Buzz Baum. Comparative rnai screening identifies a conserved core metazoan actinome by phenotype. *The Journal of cell biology*, 194(5):789–805, 2011.

[18] Y. Benjamini and Y. Hochberg. Controlling the false discovery rate: a practical and powerful approach to multiple testing. *Journal of the Royal Statistical Society Series B*, 57:289–300, 1995.

[19] KG Guruharsha, Jean-François Rual, Bo Zhai, Julian Mintseris, Pujita Vaidya, Namita Vaidya, Chapman Beekman, Christina Wong, David Y Rhee, Odise Cenaj, et al. A protein complex network of¡ i¿ drosophila melanogaster¡/i¿. *Cell*, 147(3):690–703, 2011.

[20] Thomas MJ Fruchterman and Edward M Reingold. Graph drawing by force-directed placement. *Software: Practice and experience*, 21(11):1129–1164, 1991.
